# Exploring Optimized Organic Fluorophore Search through Experimental Data-Driven Adaptive β‑VAE

**DOI:** 10.1021/jacsau.5c00052

**Published:** 2025-06-30

**Authors:** Yuzhi Xu, Yongrui Luo, Bo Li, Weikang Jiang, Jinyu Zhang, Jiangbo Wei, Hanzhi Bai, Zhiqiang Wang, Jiankai Ge, Ruiming Lin, Zehan Mi, Haozhe Zhang, Yifeng Tang, Michael S. Jones, Xiaotian Li, John Z.H. Zhang, Cheng-Wei Ju

**Affiliations:** † Department of Chemistry, 5894 New York University, New York, New York 10003, United States; ‡ Shanghai Frontiers Science Center of Artificial Intelligence and Deep Learning and NYU-ECNU Center for Computational Chemistry, NYU Shanghai, Shanghai 200062, P. R. China; § Key Laboratory of Organofluorine Chemistry, Shanghai Institute of Organic Chemistry, Chinese Academy of Sciences, Shanghai 200032, P. R. China; ∥ QuanMol Tech, Inc., San Carlos, California 94070, United States; ⊥ State Key Laboratory and Institute of Elemento-Organic Chemistry, College of Chemistry, 428705 Nankai University, Tianjin 300071, P. R. China; # Department of Chemistry and Department of Biological Sciences, 37580 National University of Singapore, Singapore 117544, Singapore; ¶ Department of Electronic Engineering, 12474 Shanghai Jiao Tong University, Shanghai 200240, P. R. China; ∇ Department of Electrical Engineering and Computer Science, 1782 Florida Atlantic University, Boca Raton, Florida 33431, United States; ○ Chemical and Biomolecular Engineering, 14589 University of Illinois at Urbana−Champaign, Urbana, Illinois 61801, United States; ⧫ Pritzker School of Molecular Engineering, 2462 The University of Chicago, Chicago, Illinois 60637, United States; †† Faculty of Synthetic Biology, Shenzhen University of Advanced Technology, Shenzhen 518055, P. R. China

**Keywords:** molecular modeling, optimization, inverse molecular design, molecules, optical properties, fluorescence

## Abstract

Designing organic fluorescent molecules with tailored optical properties has been a long-standing challenge. Recently, statistical models have opened new avenues for tackling this problem. Inverse design has attracted considerable attention in organic materials science; however, most existing approaches focus on arbitrary design or theoretical properties. Here, we introduce a strategy that enables the direct optimization of specific experimental properties during the inverse design process. Our method employs an adaptive β-variational autoencoder (adaptive β-VAE) combined with a latent vector-based prediction model. By dynamically tuning the Kullback–Leibler divergence scaling factor (β) and employing a separate training strategy, we enhance both the robustness of the generator and the diversity of the generated molecules. We demonstrate that latent vectors from the adaptive β-VAE serve as powerful inputs for downstream prediction models of experimental properties, such as fluorescence energy and quantum yield. Our optimized search framework for organic fluorescent materials—guided by gradients in latent space and validated by newly synthesized molecules sampled from optimal regions in the high-dimensional space—shows strong potential for broader applications in the design of diverse organic materials.

## Graphical abstract

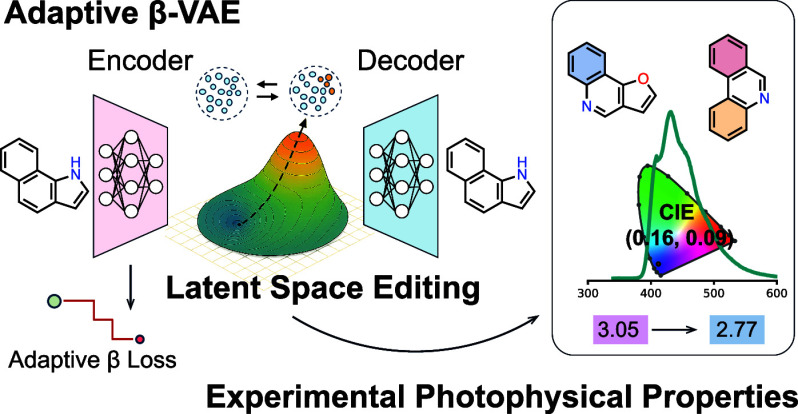

## Introduction

The design of small-molecule organic fluorophores has become a central focus in biological research and material science due to the advent of fluorescence-based applications.^1–4^ Despite this interest, the controlled synthesis of fluorophores remains challenging because of the intricate relationship between structure and properties.^5–7^ Traditional first-principles calculations offer a partial solution; however, they often fail to balance computational speed with accuracy and can only work on limited properties.^8–10^ Recent advances in machine learning (ML) have provided alternative pathways for predicting the optical properties of organic materials (Figure 1A).^11–17^ For instance, the ChemFluor data set reported by us served as the basis for our reported ML model for photophysical property prediction.^11^ Similarly, Joung et al. utilized a deep learning framework to predict a range of optical properties.^12^

**Figure 1:**
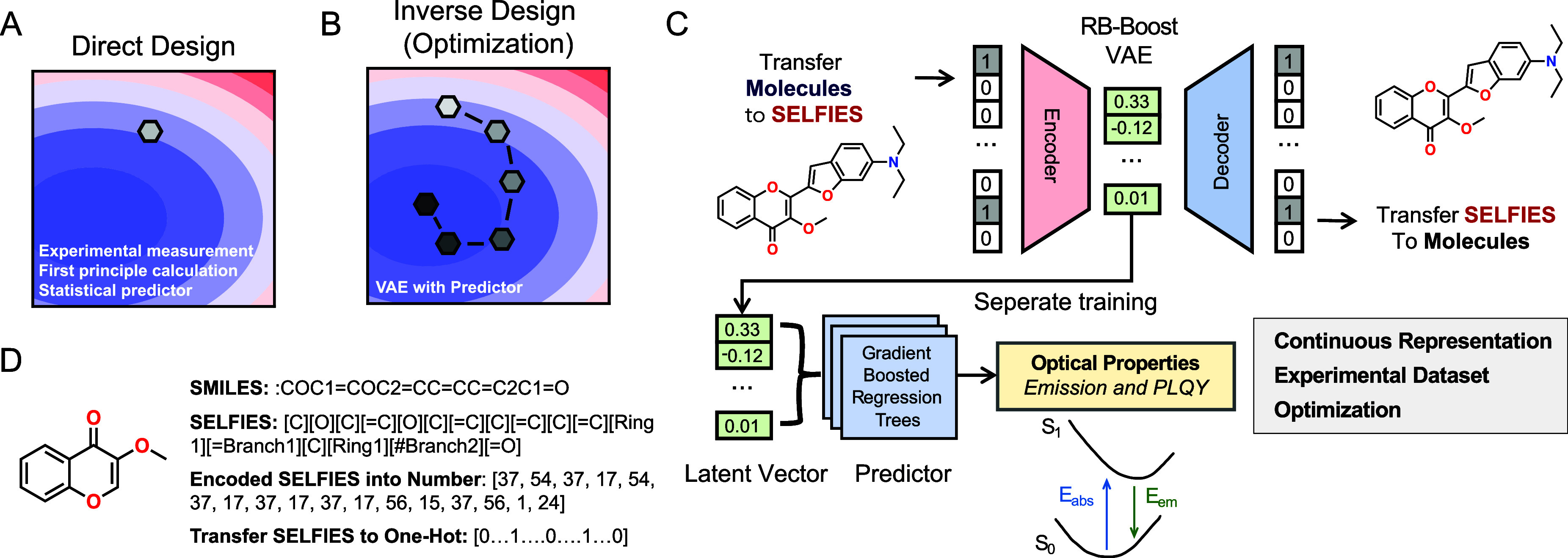
Schematic overview of organic material optimization. (A) The schematic for direct design. (B) The schematic for inverse design by searching for targeted molecules. (C) Overview of the methodology for optimized materials search developed in this study. (D) Diagram for various molecular representations.

The success of statistical models raises the possibility of inverse design and the targeted search for optimized compounds (Figure S4).^18–21^ The challenge of inverse design with predictive models for organic materials comes from the reliance on molecular descriptors, which translate molecular structures into machine-readable formats.^22,23^ This translation is unidirectional, preventing the reconstruction of molecular architectures from descriptors alone, thus limiting the scope for reverse engineering. Graph neural networks (GNNs) have shown promise in both predictive modeling and, more recently, inverse design. Nonetheless, due to their limited receptive fields and higher data requirements, fingerprint-based models remain advantageous for capturing global molecular features and enabling data-efficient training on experimentally derived data sets.^24,25^ Additionally, the discrete nature of these variables (such as molecular fingerprints) complicates the computation of gradients during optimization, posing a barrier to the seamless application of conventional optimization techniques.^26,27^ In response to these challenges, various generator architectures have garnered substantial interest.^28–30^ Early work by Aspuru-Guzik et al. on a SMILES-based variational autoencoder (VAE) opened avenues for optimized compound searches, albeit limited to small molecules.^31,32^ Moreover, the generator has been explored in ML-assisted material design as well but concentrates either on arbitrary design or theoretical properties.^33,34^

Here, we questioned whether the search for optimized compounds with specific experimental properties in materials science can also be achieved through an integrated generator-predictor framework (Figure 1B). This approach, however, presents several challenges that impede large-scale exploration. Primarily, the combination of generation and prediction tools has predominantly focused on properties derived from quantum chemical computations due to the limited scarcity of experimental data sets.^35–37^ The limited size of experimental data sets will compromise the generator’s efficacy. Additionally, this integration typically necessitates cotraining of the decoder and predictor.^32^ Lastly, predicting experimental properties—such as fluorescence wavelengths, photoluminescence quantum yield (PLQY) in organic fluorophores, power conversion efficiencies (PCEs) in organic photovoltaics (OPVs), and charge carrier mobility in organic field-effect transistors (OFETs)—proves substantially more difficult than computational attributes due to the multifaceted influences in real-world experimental conditions.

To answer these questions, we developed a workflow leveraging an adaptive β-VAE and a predictor to directly optimize organic fluorophores on a high dimensional space fitted from experimental energies (Figure 1C). SELFIES, a robust and standardizable molecular string representation, was utilized for reliable encoding in a one-hot format (Figure 1D and Method S1.1.1). The application of this encoding is to reduce the model’s dependency on learning syntax alongside molecular structure, thereby minimizing syntax-related errors during generation. We train the generator and predictor separately and thus make the data fusion in the generator become possible. Dynamic tuning of the scaling factor of the Kullback–Leibler divergence (KL divergence), β, which regulated the strength of the regularization, can generate a more flexible latent space representation and improve the decoder’s reconstruction ability. Utilizing the latent vectors from this adaptive β-VAE, we constructed a prediction model for the photophysical properties, including PLQY and emission energy within the error of quantum mechanical precision (∼0.13 eV). Then, we visualize the high-dimensional space to confirm the possibility of target molecular optimization. Experimental validation with newly synthesized molecules sampled from optimal regions of high-dimensional space successfully confirms the feasibility of our generator and predictor. Applying our method in a fluorophore skeleton, we synthesized a new compound with bright blue emission, showcasing our strategy’s potential for material discovery. Our workflow proves the feasibility of inverse design achieved through target optimization and signals a transformative approach to diverse organic material design.

## Results and Discussion

### Adaptive β-VAE for Molecular Reconstruction

Traditional autoencoders (AE) focus on compressing and reconstructing data but lack control over the latent space, limiting their usefulness for generating diverse molecular structures. VAE, on the other hand, provides structured latent spaces that are ideal for molecular generation by introducing a KL divergence term. However, this structure can sometimes overconstrain the model, reducing reconstruction efficiency. To address this, β-VAE was introduced, adding a scaling factor, β, before the KL divergence term (Figure 2A). Adjusting β provides more flexibility: lower β values reduce the influence of KL divergence, allowing for higher reconstruction accuracy, while higher β values increase the regularization effect, encouraging diverse generation. β-VAE allows for control over the balance between reconstruction accuracy and diversity. However, a fixed β may still be suboptimal, as different stages of training demand varying levels of regularization.

**Figure 2:**
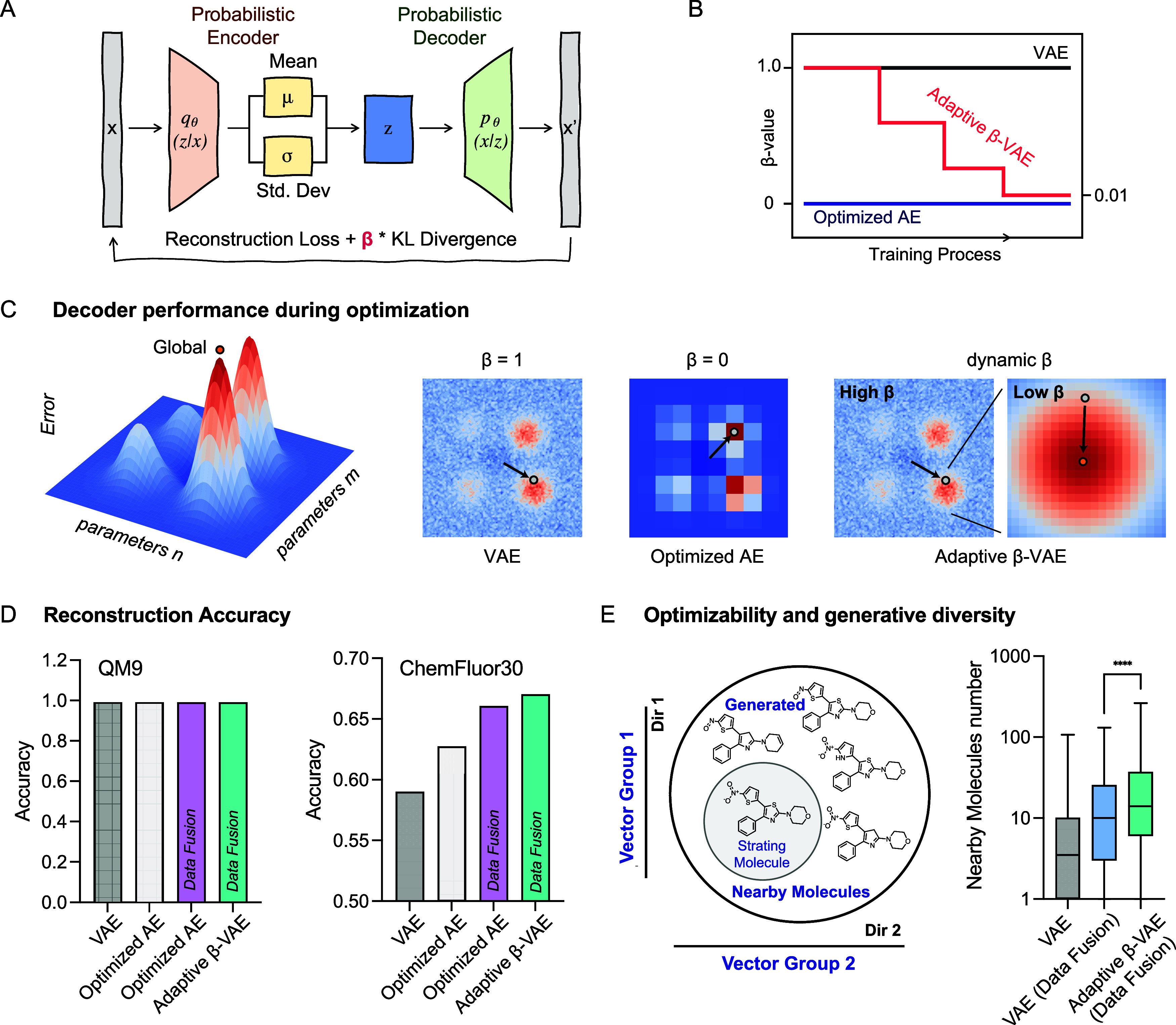
Enhancing molecular diversity and accuracy through adaptive β-VAE. (A) Schematic for the VAE and β-VAE. (B) The change of β value during the training process in VAE, optimized AE, and adaptive β-VAE. (C) Conceptual illustration of the optimization process under different β settings. (Left) Error landscape with respect to hyperparameters n and m. The goal of tuning the β value is to guide the model toward the global optimum (marked in orange). (Middle) Latent space representations under different KL divergence weights: standard VAE (β = 1) encourages structured diversity but may lead to noisy representations and convergence to a local minimum; AE (β = 0) drives the model toward a global minimum in terms of reconstruction error but sacrifices diversity and may ignore the true global optimum. Adaptive β-VAE transitions from global exploration (high β) to localized accuracy (low β), effectively balancing both objectives. Heatmaps represent reconstruction error. Arrows indicate training trajectories. (D) Evaluation of the reconstruction accuracy of different AE variants across various data sets. (E) Adaptive β-VAE enhances diversity in molecular generation. The box plot shows the capability of adaptive β-VAE in generating viable molecules from latent vector interpolations, while only molecules generated by disturbing a randomly selected and consistent subset of vectors are used for the counts.

To tackle this challenge, we propose a β-VAE variant, named adaptive β-VAE. Our approach uses a dynamic β that changes over the course of training: we start with a high β to build a globally diverse latent space and then gradually decrease β, allowing the model to emphasize local reconstruction accuracy (Figure 2B, see Methods S1.1.2 and S1.1.3 for details). This adaptive strategy enables the model to explore a broader range of possibilities at early stages and converge to accurate solutions later, striking a balance between AE’s reconstruction focus and VAE’s generative flexibility (Figure 2C). This approach is particularly effective for specific data types, such as chemical small molecules, where both local detail and global diversity are essential.

Cotraining prediction and generative models have been common in molecular generation; however, this approach is limited by experimental data sets, which are often small in size, restricting the model’s generative diversity. To overcome this, we opted for separate training of the generator and predictor, allowing each model to specialize without data set limitations. Additionally, we incorporated diverse molecular scaffolds through data fusion, enriching our data set with compatible molecules per established protocols (Method S1.1.4). This strategy broadens latent space sampling and enhances generative diversity, addressing real-world challenges in molecular generation.

We first validated our model on the QM9 data set with three VAE variants (β = 1 VAE; β = 0, optimized AE; and adaptive β-VAE), achieving a high reconstruction rate (>98%) across all variants (Figure 2D and Table S1). When our strategy was applied to a more challenging data set with larger fluorescent molecules, ChemFluor30 (a subdata set of ChemFluor with molecules smaller than 30 heavy atoms), the adaptive β-VAE with data fusion showed a clear performance improvement, raising reconstruction rates from 59% to 67%, a relative increase of ∼13% (Figure 2D). Ablation experiment confirmed the impotence of scheduling strategy for the β value in adaptive β-VAE (Table S2). This dual strategy not only increase the reconstruction accuracy but also enhanced the representation of diverse molecular characteristics.

We then evaluated the enhanced adaptive β-VAE and the VAE by perturbing a subset of latent vectors to generate molecules (Figures 2E and S5). The adaptive β-VAE demonstrated superior performance, generating an average of 8.2 times more total distinct molecules with a broader chemical feature set, indicative of a more complex chemical space encapsulated during model training (Figure 2E). This contrasted with the original VAE, which tends to generate more similar structures. Moreover, the adaptive β-VAE facilitated the generation of transitional molecular structures through interpolation between two selected latent vectors (Figures 2E and S6). Despite some resulting nonviable molecules, the majority of these intermediate structures were coherent and synthesizable, emphasizing the strength of our strategy in refining the VAE architecture to generate a wide range of diverse molecules.

### Predictor Based on GBRT with the Latent Vector for Experimental Optical Properties

With the establishment of the generator, we move to the prediction model. To adapt our VAE for chemical property prediction, we train the predictor separately using the latent space learned from the *ChemFluor30* data set, which contains experimentally measured photophysical properties. This approach diverges from the conventional joint training approach, which often restricts chemical diversity.^32^ Our investigation prioritizes emission energies—key optical properties for organic emitters. We adopt the Gradient Boosting Regression Tree (GBRT), lauded for its predictive precision in our prior research (Method S1.1.5 and Tables S3 and S4). The model results in a mean absolute error (MAE) of 0.128 eV for unseen molecules in different solvents using latent vectors as the input, surpassing TD-DFT accuracy (∼0.20 eV), and is sufficient for utilizing in virtual screening (Figure 3A and Table S5)^38–42^ A similar MAE of 0.124 eV was obtained from one-hot SELFIES as input indicating the high fidelity of the latent vector generated from SELFIES. Furthermore, the model successfully reproduces the trends of specific molecules in different solvents (Figures 3B and S7). To externally validate the model, we test it on a data set of NDI, Rhodamine, and Coumarin molecules previously used as benchmarks. The model maintains a strong performance with an MAE of 0.20 eV, comparable to TD-DFT accuracy (Figure S8). Utilizing T-distributed stochastic neighbor embedding (t-SNE) visualizations, we observe the cluster of various structures such as Rhodamine and BODIPY derivatives (Figure 3C). Meanwhile, the analogous distributions between latent vectors and SELFIES prove that they are high-fidelity predictors, while the distinct from ECFP4 suggests their uniqueness (Figure S9). Furthermore, based on the predictor, we confirm that the molecules generated by adaptive β-VAE exhibit a greater diversity in their predicted emission energies (Figure S10).

**Figure 3:**
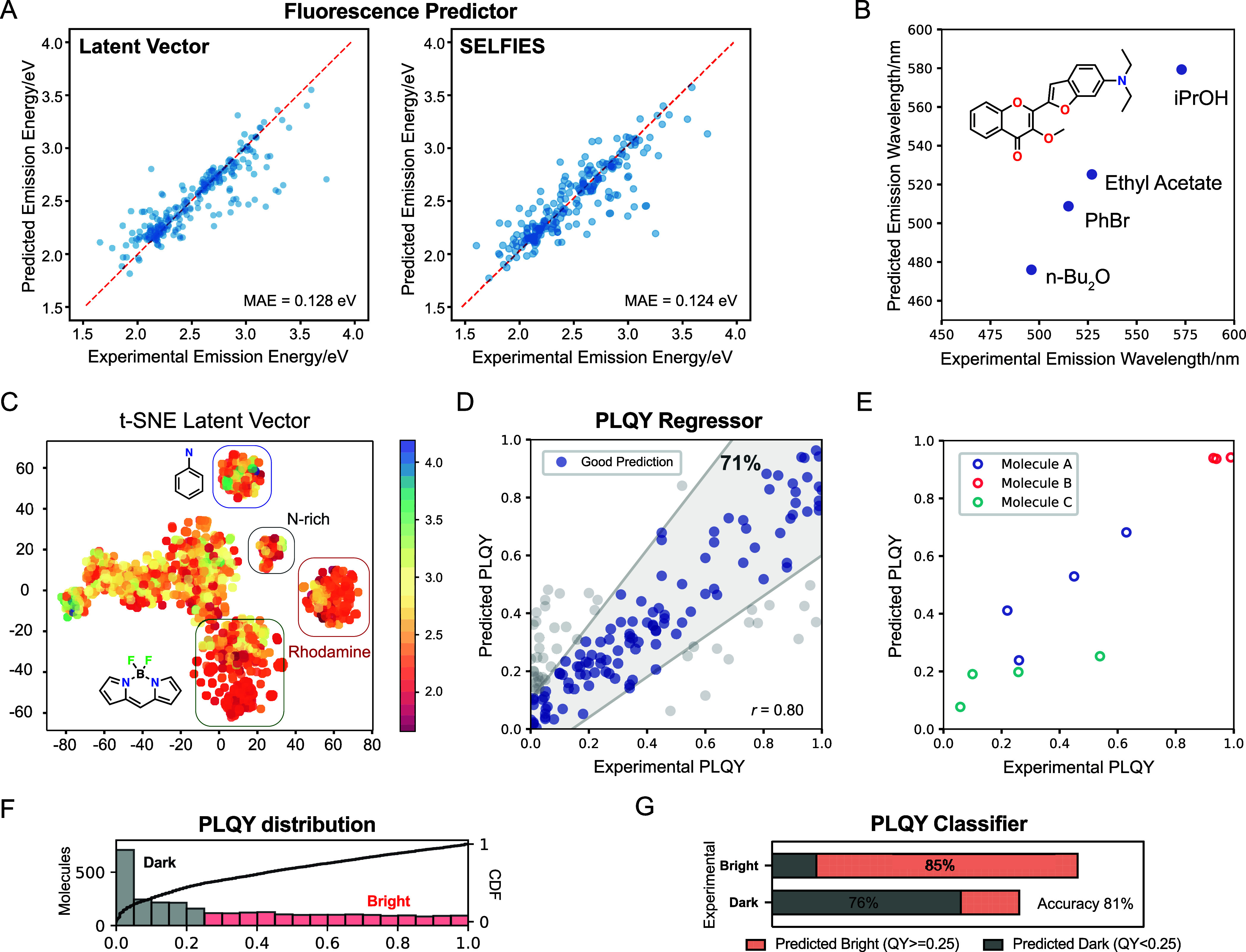
Latent vector-based predictor for optical properties. (A) The predictor for the emission energy based on GBRT with latent vector (left) or SELFIES (right) well reproduces the fluorescence in the test set for unseen molecules with different solvents. (B) The fluorescence wavelength of one typical molecule in the test set in various solvents. (C) t-SNE of latent vectors obtained from adaptive β-VAE. Colors indicate the emission energies (eV). (D) The regressor for the PLQY based on GBRT with the latent vector as input can reproduce the brightness for unseen molecules. (E) PLQY for selected molecules in the test set under various solvents. (F) The distribution of PLQY in the data set. 0.25 is set as the threshold for bright and dark molecules. (G) The prediction performance of PLQY classifier with the latent vector as input.

Furthermore, we also assessed PLQY predictions within the latent space. PLQY is one of the most critical factors affecting the fluorescence intensity of organic fluorescent materials, yet attempts at its prediction remain limited. Our regressor achieves reasonable accuracy for unseen molecules across various solvents (*r* = 0.80, Figure 3D), making it suitable for prescreening fluorophore candidates. To study the prediction accuracy for real-world problems, we define an accurate PLQY prediction if the absolute error is less than 30% of the true value plus 0.1, an empirically chosen threshold that reflects typical practical tolerance and experimental uncertainty.[ref] Over 70% of unseen molecules can be accurately predicted, outperforming TD-DFT-based estimations.^43,44^ It is also important to note that TD-DFT cannot easily or broadly estimate the PLQY. Only a few studies have attempted such calculations under specific physical assumptions, and these approaches are limited to a range of molecular systems. Additionally, our model can also well reproduce solvent effects (Figure 3E). Considering the distribution of PLQY and real-world situations, we apply 0.25 as a threshold to classify the bright and dark molecules (Figure 3F).^45–47^ Our classifier discerns between bright and dark materials with an accuracy of 0.81, rendering it suitable for practical predictive applications (Figure 3G).

### Synthesis Validation of the Framework

Based on the demonstrated performance of our generator and predictor, we have utilized vector group tuning to visualize the high-dimensional space in a 3D plot, facilitating precise structural adjustment and exploration (Methods S1.1.6 and S1.1.7). We applied our approach with molecules shown in the center of Figure 4A, where the manipulation of latent vectors yielded diverse molecules with predicted emission energies ranging from 1.95 to 2.45 eV (Figure 4A for model based on adaptive β-VAE and Figure S11 for optimized AE). To validate the reliability of the predicted fluorescence energy in the generated high-dimensional space, we employed Semiempirical Tight Binding, GFN2-xTB, a semiempirical quantum mechanical method to estimate the HOMO–LUMO gap of several molecules with a similar skeleton generated in this high-dimensional space (Figure S12).^48^ Molecules with similar skeletons are selected here for the computational validation since we want to minimize the structure diversity that increases the complexity and difference between computational and experimental properties. The correlation further supports the validity of our approach (Figure 4B). Although it needs to be recognized that (1) semiempirical methods are not accurate and (2) calculated H-L gap only reflects the electronic structure in the ground state while emission is highly related to the excited state, we rationalize that molecules with a similar skeleton should at least have similar trend between H-L gap and fluorescence wavelength. This localized optimization highlights our approach’s potential in editing molecular structures and properties, confirming its utility in precision design.

**Figure 4:**
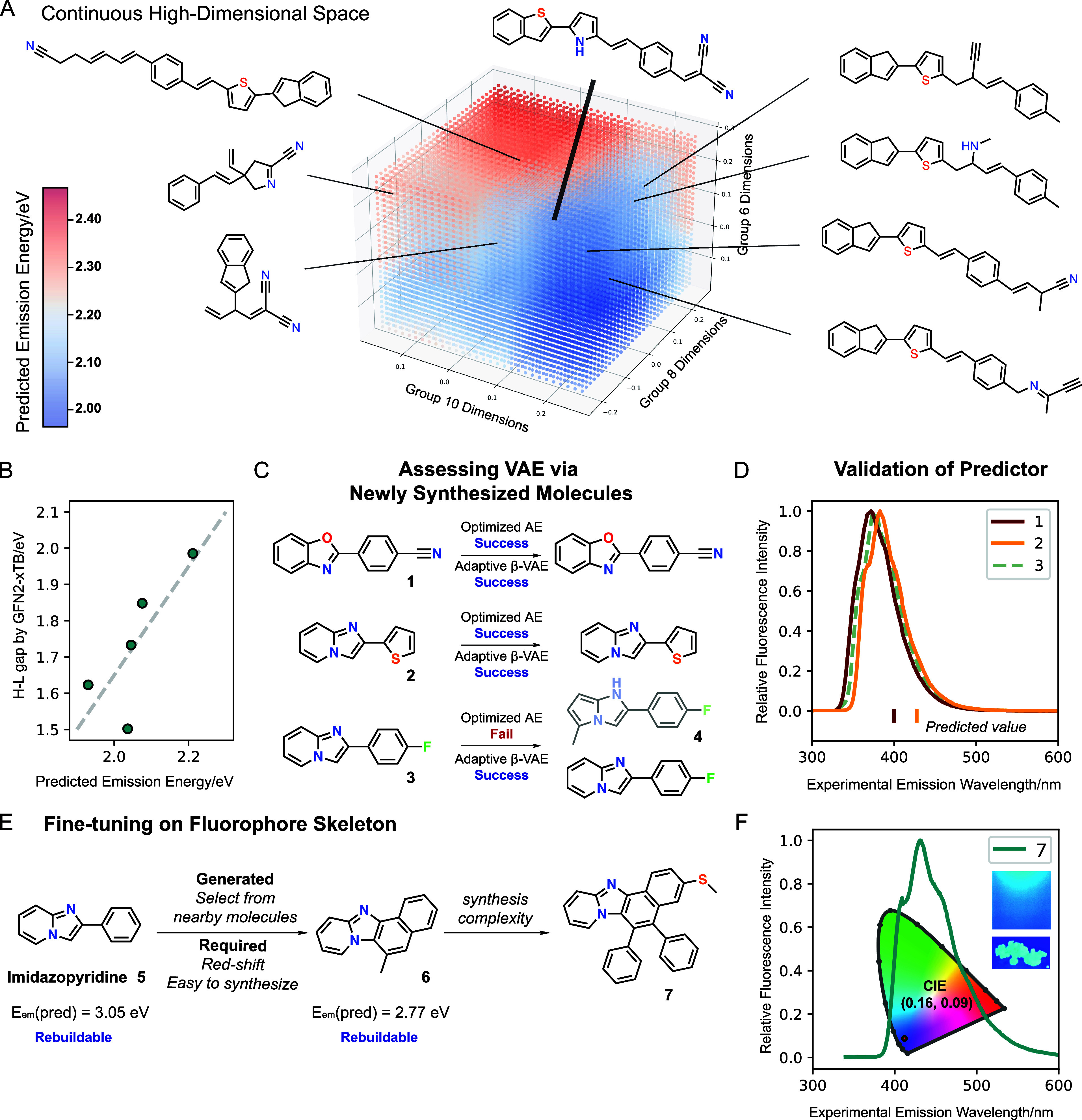
High-dimensional latent space analysis and synthesis validation. (A) Visualization and analysis of the continuous high-dimensional space, indicating potential for optimization. (B) Correlation between the HOMO–LUMO gap calculated by GFN2-xTB and the predicted emission energy of molecules with similar backbone sampled from high-dimensional space. (C) External validation of RB-Boost VAE using uncharacterized synthesized molecules. (D) Comparison of experimental fluorescence spectra with predicted emission energies for uncharacterized molecules, illustrating prediction accuracy. (E) Editing on the fluorophore skeleton (imidazopyridine) by exploring nearby molecules and controlling synthesis complexity. (F) The fluorescence spectrum of molecule **7**.

To further corroborate our strategy’s efficacy, we synthesized and analyzed novel molecules. Initially, derivatives of benzoxazole and imidazopyridine (**1**–**3**) were analyzed by using the optimized AE and adaptive β-VAE. Compounds **1** and **2** were successfully reproduced by both methods. However, compound **3** underwent a transformation to 5-methyl-1*H*-pyrrolo­[1,2-*a*]­imidazole **4** with the optimized AE but was accurately reproduced by the adaptive β-VAE (Figure 4C). Later, to evaluate the performance of the predictor, we characterized their fluorescence spectra in CH_2_Cl_2_ (Figure 4D). Although the absolute error is around 0.20 eV, the model accurately reflected the emission trend for **1** and **2**, which possess a similar biaryl backbone. Following this initial validation of the generator and predictor, we investigated the utility of our strategy in optimized compound searches and molecular editing. Due to the complexity introduced by the high-dimensional latent space, we centered our exploration on the nearby molecules of imidazopyridine derivative **5** (Figure 4E). We choose molecule **6** with an extended π-system, for its plausible structure and predicted red-shifted emission compared with **5** (3.05 eV to 2.77 eV). Considering synthetic feasibility and our laboratory’s compound library, we synthesized **7** based on the backbone of **6**. The photophysical characterization of **7** revealed its bright blue emission with a CIE coordinate (0.16, 0.09), indicating its potential as a blue OLED emitter (Figure 4F).^49^

## Conclusions

In summary, we successfully leveraged the latent vector space to enable optimized molecule generation with experimentally relevant properties through a combination of adaptive β-VAE and a predictor. Specifically, we applied adaptive β-VAE, which employs a dynamically tuned scaling factor, β, for KL divergence to regulate the strength of regularization. This tuning of β enabled a flexible latent space representation, enhancing both the reconstruction accuracy and molecular diversity. Unlike traditional workflows, our predictor actively informed the selection of latent vectors, optimizing the search for “dream molecules” with tailored properties. We confirmed the practicality of our method in searching for optimized compounds by (1) the evaluation of the predictor performance, (2) visualization of the latent space with predicted emission energy validated by semiempirical quantum mechanical methods, and (3) experimental validation of synthesized molecules. Using a fluorophore skeleton as an example, we designed and synthesized compound **7**, which exhibited bright blue emission, demonstrating the feasibility and potential of our strategy in materials discovery.

This streamlined workflow not only enables editing of molecular properties for optimized compounds but also heralds a new era of material design with promising applications in the development of OLEDs, OPVs, and OFETs. Despite its success, the current approach has limitations, including the reliance on relatively small experimental data sets and the need for improved predictors for complex experimental properties. Furthermore, systematically benchmarking AI-driven reverse design against heuristic-driven expert strategies would be valuable for understanding the full potential of these data-driven approaches. Future work will address these challenges by expanding experimental data sets, integrating diffusion model with advanced neural network predictors, and exploring multitask learning frameworks.^50–55^ These efforts will further enhance the robustness, accuracy, and versatility of AI-driven molecular design, paving the way for transformative applications across materials science and biotechnology.

## Methods

### Variational Autoencoder

The VAE, developed by Diederik P. Kingma and Max Welling, reframes statistical inference issues as optimization problems.^56^ In a VAE, the input data is sampled from a parametrized distribution, and the encoder and decoder are trained together to minimize the reconstruction error between the parametric and true posterior distributions.

When the model receives input x, the encoder compresses it into the latent space. The decoder then takes information sampled from this space to produce an output x as similar as possible to x. However, rather than encoding an input as a single point in the latent space, the VAE represents it as a distribution over this space. Thus, the encoder returns a distribution over the latent space instead of a single point. A regularization term is added to the loss function over this distribution to ensure a well-organized latent space conducive to the generative process.

The VAE’s primary mechanism involves maximizing the evidence lower bound (ELBO). The ELBO is formulated as follows

$$ELBO=E_{q_{\phi }\left(z|x\right)}\left[\log p_{\theta }\left(x|z\right)\right]-KL\left[q_{\phi }\left(z|x\right)|p\left(z\right)\right]$$

Here, *q*_ϕ_(*z*|*x*) represents the approximate posterior distribution of the latent space learned by the encoder, *p*_θ_(*x*|*z*) is the conditional probability distribution of the data generated by the decoder, *p*(*z*) is the prior distribution of the latent space, and KL denotes the Kullback–Leibler divergence, measuring the divergence between two distributions. By maximizing the ELBO, the VAE aims to improve the quality of data reconstruction while maintaining an effective organizational structure in the latent space. We use the framework of VAE, as shown in Figure S1.

### Data Sets

Two data sets have been applied in this work to construct a generative model: (1) *QM 9_sub*, which contains 25000 small organic molecules obtained randomly from QM9 data set.^57^ Molecules in the data set consist of H, C, O, N, and F and contain up to 9 heavy atoms. As shown by the distribution in Figure S2A, about 80% of the molecules contain 9 atoms. Compounds in this data set is considered small organic molecules; (2) ChemFluor is composed of more than 4300 experimental solvated organic fluorescent materials (around 3000 distinct compounds) and 11,000 data (λ_abs_, λ_em_, and PLQY).^11^ Most of the molecules contain more than 20 atoms (Figure S2b). A subdata set of ChemFluor named *ChemFluor30*, which contains 2280 molecules with atomic number less than 31, has been used in this work. 80% of the data set is randomly selected as used as the training set. 10% of the data set is used as the validation set and 10% of the data set is used as the test set. The percentage of the molecules that successfully reproduced by VAE is used to evaluate the performance of various models. To comprehensively analyze the rebuild rate of different decoders and encoders, we evaluate VAE, optimized AE, and adaptive β-VAE in both QM9_sub and ChemFluor30 data set.

### Optimized AE and Adaptive β-VAE

In our research, we present a variant AE, termed optimized AE in our work, which adapts the traditional ELBO by excluding the KL divergence. This alteration allows the model to primarily focus on learning the latent distribution without diverging toward generating novel molecular structures.

We also developed a variant of the β-VAE, termed adaptive β-VAE, which adapts the traditional ELBO by modifying the KL divergence. This alteration allows the model to primarily focus on learning the latent distribution without diverging toward generating novel molecular structures. To be more specific, in the basic β-VAE, the parameter modifies the objective by introducing a β-term to balance the reconstruction and regularization

$$L_{\beta }\left(\theta ,\Phi ;X\right)=E_{q\phi \left(ẐX\right)}\left[\log p_{\theta }\left(\mathrm{X̂}Z\right)\right]-\beta D_{KL}\left(q_{\phi }\left(ẐX\right)\hat{\mathrm{P̂}\left(Z\right)}\right)$$

The choice of a fixed β value is known to influence the learned representation. A larger β emphasizes disentanglement and a smoother latent space at the expense of reconstruction fidelity, while a smaller β prioritizes data fidelity over latent regularization. Although a single β-value is conceptually simple, it cannot adapt to the evolving needs of the training process. Early in training, encouraging a well-structured latent space can prevent representations from collapsing into narrow regions. Later in training, allowing more focus on reconstruction can refine the learned distributions and ensure high-quality decoding.

Therefore, we use an adaptive strategy to modify this process: An exponential decay schedule is one of the simplest adaptive strategies. Suppose β_start_ is the initial β-value, β_end_ is a lower bound, and ρ∈(0,1) is a decay rate. At epoch t, we define β^(*t*)^ = max­(β_end_,β_start_·ρ^*t*^). In early epochs, β^(*t*)^≈β_start_, which is typically chosen to be ≥1 to ensure a well-regularized latent space. As the training progresses, β^(*t*)^ smoothly decreases, shifting the balance toward more accurate reconstructions.

While exponential decay is effective and simple, other heuristics can be employed:

Linear decay: β decreased linearly over epochs until reaching β_end_

Piecewise scheduling: using a high β during the initial *T*_switch_ epoch and then abruptly lowering it thereafter.

$$\beta^{\left(t\right)}{=\beta }_{high}\text{if}t<T_{switch},\beta^{\left(t\right)}{=\beta }_{low}\text{if}t\geq T_{switch}$$

Performance-based adjustment: monitoring KL divergence and reconstruction loss during training and adjusting β accordingly. For instance, if the KL term becomes too small, β is temporarily increased; if reconstruction lags, β is decreased. These alternatives provide flexibility and can be tailored to specific data sets or training objectives. In our work, ρ∈(0,1), we select the 0.95, and therefore, the final loss can be expressed as

$$L_{\beta }\left(\theta ,\phi ;x\right)=E_{q_{\phi }\left(z|x\right)}\left[\log p_{\theta }\left(x|z\right)\right]-\beta D_{KL}\left(q_{\phi }\left(z|x\right)|p\left(z\right)\right)$$

### Data Fusion

We also have enhanced the diversity and recognition capabilities of our molecular data set by incorporating a subset of molecules from the *ChemFluor30* data set and expanding it through similarity-based augmentation using the PubChem database. Furthermore, to enrich our data set with high-quality chemical structures, we have integrated data from the Joung et al.^12^ Following a stringent selection process that filters molecules based on a maximum atom count criterion of 31 atoms, we combined the data sets. The resultant augmented database contains a total of 5310 molecules, significantly broadening the chemical space for the training of our models.^4^ This methodological enhancement facilitates the learning of a generalized molecular representation.

## Supplementary Material



## Data Availability

We express our sincere gratitude to Joung et al. for generously sharing their open access data set.^12^ The data and data sets utilized in this manuscript are derived from prior publications and the PubChem database, specifically refs 11 and 12 . All the data and code in our work can be found on the CodeOcean platform at https://codeocean.com/capsule/7686798/tree/v1 and are publicly available.

## Notes

General experimental procedures, materials, and instruments; detailed computational methods; predictor benchmarking and discussion; ^1^H and ^13^C NMR spectra for all compounds; metrics for VAE reconstruction; performance of the adaptive β-schedule; benchmarking results for latent-space predictors; 10 × 5-fold cross-validation statistics; comparisons across emission energy prediction methods; molecular encoding workflow; molecular design pathways; diversity analysis; latent space visualizations; solvent-dependent fluorescence data; and external set validation (PDF)
