# Supplementary material for: Exploring Optimized Organic Fluorophore Search through Experimental Data-Driven Adaptive β‑VAE
Source: JACS Au. 2025 Jun 30;5(7):3082–91. doi: 10.1021/jacsau.5c00052 (PMC12308382; doi:10.1021/jacsau.5c00052)
Supplement: Supplementary file 1 [file au5c00052_si_001.pdf]

Supporting Information for

Exploring Optimized Organic Fluorophore Search Through Experimental Data-Driven Adaptive β-VAE

Yuzhi Xu[1,2], Yongrui Luo[3], Bo Li[4], Weikang Jiang[3], Jinyu Zhang[5], Jiangbo Wei[6], Hanzhi Bai[7], Zhiqiang Wang[8], Jiankai Ge[9], Ruiming Lin[10], Zehan Mi[10], Haozhe Zhang[10], Yifeng Tang[10], Michael S. Jones[10], Xiaotian Li[11], John Z.H. Zhang[1,2,11], Cheng-Wei Ju[10],*

1. Department of Chemistry, New York University, New York, New York 10003, United States
2. Shanghai Frontiers Science Center of Artificial Intelligence and Deep Learning and NYU-ECNU Center for Computational Chemistry, NYU Shanghai, Shanghai 200062, P. R. China
3. Key Laboratory of Organofluorine Chemistry, Shanghai Institute of Organic Chemistry, Chinese Academy of Sciences, Shanghai 200032, P. R. China
4. QuanMol Tech, Inc., San Carlos, California 94070, United States
5. State Key Laboratory and Institute of Elemento-Organic Chemistry, College of Chemistry, Nankai University, Tianjin 300071, P. R. China
6. Department of Chemistry and Department of Biological Sciences, National University of Singapore, Singapore 117544, Singapore
7. Department of Electronic Engineering, Shanghai Jiao Tong University, Shanghai 200240, P. R. China
8. Department of Electrical Engineering and Computer Science, Florida Atlantic University, Boca Raton, Florida 33431, United States
9. Chemical and Biomolecular Engineering, University of Illinois at Urbana-Champaign, Urbana, Illinois 61801, United States
10. Pritzker School of Molecular Engineering, The University of Chicago, Chicago, Illinois 60637, United States
11. Faculty of Synthetic Biology and Institute of Synthetic Biology, Shenzhen Institute of Advanced Technology, Shenzhen 518055, P. R. China

E-mail: C.-W. Ju, chengwei.ju99@gmail.com

1. Methods

1.1. Variational Autoencoder (VAE)

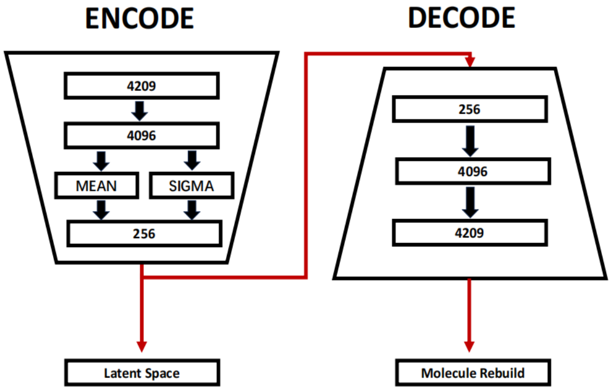

Figure S1. Framework of VAE used in this work, following the structure proposed by Kingma et al.[1]

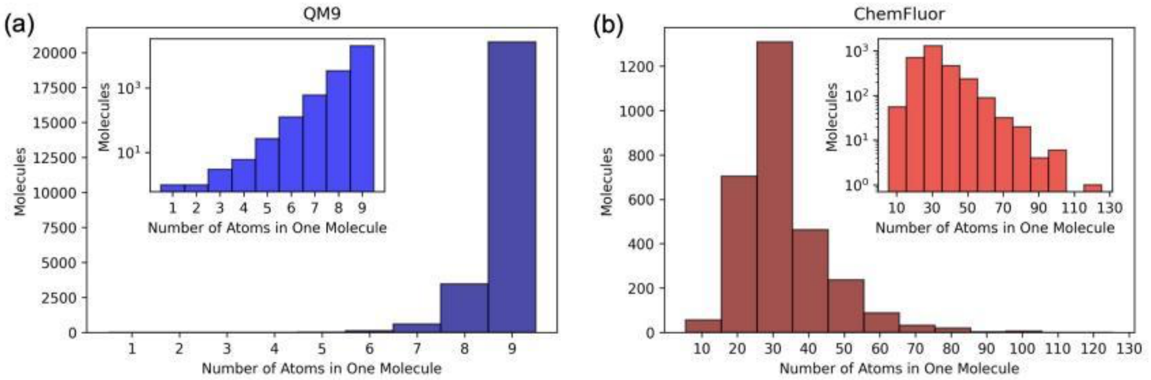

Figure S2. Distribution of atom numbers in (a) QM9 sub-dataset[2] and (b) ChemFluor dataset[3].

1.1.1. Latent vector tuning

Molecular generation in latent space is conducted through vector group tuning. We refined the tuning framework initially proposed by Gómez-Bombarelli et al. to better suit the requirements of our system[4]. Specifically, the latent vectors are divided into eight vector groups using an arithmetic sequence. Among these, the two vector groups with the greatest influence on prediction outcomes are selected. Initial statistical measures are then computed: for each of the selected vector groups, the mean and standard deviation are calculated. The mean determines the starting point, while the standard deviation defines the range of step sizes. Two sets of uniformly distributed values are generated within the defined intervals, with each vector group yielding 30 interpolation points for sample editing.

Similarly, the range of editable vector groups can be expanded to further enhance the diversity of the edited samples. As shown in Figure 4a, by expanding the generation range, three vector groups can be selected to further enhance the diversity of the edited samples. These combinations form a three-dimensional coordinate system, where each selected vector group corresponds to an axis (x, y, z).

1.1.2. Molecular generation for the emitter

We started with molecule A and transformed it into molecule B using a series of methods. Initially, we set a range for each element of random arrays, roughly between 5e-3 and 1e-1. We created 400 different sets of random vectors by introducing various random noises while starting with an initial random vector. To add more variety, we used a 'multiplicative scaling' technique to change molecule A's properties. This involved multiplying molecule A's properties and the random vectors by 0.1 times 'k' (where 'k' ranges from 0 to 10) and then adding them together, resulting in 121 combinations for each random vector.

To make sure the molecules were structurally stable, and the synthesis process was manageable, we developed some screening rules based on our chemical expertise. These rules included removing certain types of molecular structures, getting rid of straight chains, and keeping the size of the rings between 5 and 9 atoms. After going through multiple rounds of generating and screening, we successfully created 243 different molecules.

1.2. Predictor

1.2.1. Gradient Boosted Regression Trees (GBRT)

GBRT is a flexible non-parametric statistical learning technique for classification and regression.[5,6] It is one of the most effective machine learning models for predictive analytics. As an ensemble learning algorithm, GBRT combines weaker base learners (decision trees) into a stronger learner using an iterative scheme.

The main idea of GBRT is a search of a target function (F) of input variables (xi) to reach output variables (yi) by minimizing a cost function (L[yi, F(xi)]), which was originated by Breiman and further developed by Friedman and is referred to as the gradient boosting machines (GBM). GBM starts with an initial F0 that minimizes the sum of the loss function.and updates it in every iteration based on weighted sums of functions (hm) generated by base learners.It updates hm based on the training set xi, rim where each gradient component is generated by and the multiplier (γm) is obtained by minimizing the sum of the new loss function. The most important two hyperparameters of GBRT are klearn, the rate the contribution of each tree is shrunk, and ntree, the number of decision trees in the ensemble. Large klearn and ntree usually result in strong performance because GBRT is fairly robust to overfitting. The GBRT model is very good at handling tabular data with numerical features, or categorical features with fewer than hundreds of categories. Unlike linear models, the GBRT model is able to capture non-linear interaction between the features and the targe.

1.2.2. Input for the prediction model and performance evaluation

In this work, we use the latent space extracted from the VAE model as the input for training the prediction model. The dataset division is kept the same as mentioned for the VAE model. 80% of the dataset is randomly selected as used as the training set. 10% of the dataset is used as a validation set and 10% of the dataset is used as the test set.

The accuracy of the predictor, evaluated by mean absolute error (MAE), Root Mean Square Error (RMSE) and coefficient of determination(r);

1.3. Descriptors

1.3.1. SELFIES

SELFIES (SELF-referencIng Embedded Strings) is a 100% robust molecular string representation.[7] These representations are independent of the machine learning model which can be used as a direct input without any adaptations of the models. Each SELFIES corresponds to a valid molecule, and every molecule can be described as a SELFIES.

Comparing with SMILES, the model's internal memory of SELFIES stores two orders of magnitude more diverse molecules. In SELFIES, the information of molecule like branch length as well as ring size is stored together with the corresponding identifiers 'Branch' or 'Ring'. The symbol after the 'Branch' or 'Ring' means a number that is interpreted as lengths, to avoid the possibility of invalid syntactical string. SELFIES symbols are generated from SMILES using derivation rules.

1.3.2. SELFIES Matrix

In our study, we also utilized a method known as the SELFIES Matrix as the input for the VAE. We performed a simple one-hot sparse transformation on the SELFIES. Through this sparse transformation, we were able to generate the matrix that feeds into the VAE. More specifically, since SELFIES are segmented using brackets '[ ]', and their representations are finite, we can extract the maximum character limit from the entire dataset. Considering that the training dataset is significantly larger than the test dataset, we assume that the character representation in the training set can encompass that of the test set. The two dimensions in matrix represent the maximum string length and the number of characters in the training set. Based on this, we encoded our QM9/ChemFluor30 datasets separately. For ChemFluor30, the first and second dimensions are 69 and 58, respectively.

To better understand the SELFIES Matrix method, we use the molecular example "C=COC" as a simple example to illustrate it (**Fig S3**). The SMILES representation "C=COC" is first converted into the SELFIES representation "[C][O][C][=C]". Each SELFIES character is then mapped onto a one-hot encoded vector of length 58, where each vector has a single position marked as 1, corresponding to the specific SELFIES character, and all other positions are set to 0. These vectors are vertically stacked in the order they appear in the SELFIES string to form a 69x58 matrix. Each row of this matrix represents a character from the SELFIES string, and the width of 58 columns reflects the number of different SELFIES characters present in the training set.

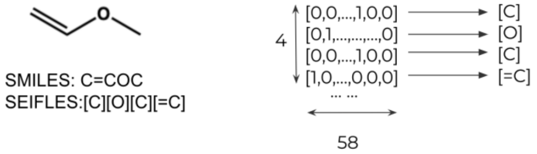

Figure S3. Process of converting the SMILES representation of a molecule, "C=COC", into its SELFIES representation "[C][O][C][=C]", and subsequently transfer into a one-hot encoded matrix for use in a VAE.

1.4. Computation details

The semi-empirical tight-binding calculations were performed in GFN2-xTB.[8] For each molecule, the geometries were generated based on SMILES information using RDKit.

1.5. Photophysical properties characterization

Photoluminescence spectra were recorded on an Edinburgh Instruments FS5 fluorescence spectrometer with a concentration around 10^{-5} mol/L in CH_2Cl_2. To reduce the fluctuation in the excitation intensity, the lamp was kept on for 1 hour prior to the experiment.

2. Supplementary Tables and Figures

Table S1. Performance of various VAE in different dataset**.**

Dataset	Model	Rebuild Rate
QM9_sub	VAE	98.5%
QM9_sub	Optimized AE	98.4%
QM9_sub	Optimized AE (Data Fusion)	98.4%
QM9_sub	Adaptive β-VAE (Data Fusion)	98.5%
ChemFluor30	VAE	59.1%
ChemFluor30	Optimized AE	63.4%
ChemFluor30	Optimized AE (Data Fusion)	66.1%
ChemFluor30	Adaptive β-VAE (Data Fusion)	67.2%

Table S2. Performance of Adaptive β-VAE with various β scheduling strategy**.**

β_{start}	β_{end}	ρ	Rebuild Rate
**1**	**0.01**	**0.95**	**67.20%**
0.5	0.01	0.95	66.67%
1	0.01	0.5	65.05%
1	0.3	0.95	65.05%
1	0.5	0.1	65.05%

Table S3. Performance of different prediction model in this work**.**

Model	MAE (eV)	RMSE (eV)	R^2	*r*
**GBRT**	**0.128**	**0.202**	**0.738**	**0.859**
XGBoost	0.136	0.209	0.720	0.854
KRR	0.235	0.309	0.394	0.650
SVR	0.144	0.209	0.723	0.853
DNN	0.157	0.242	0.627	0.810
ANN(MLP)	0.208	0.338	0.276	0.675

We employed several representative machine learning models, including eXtreme Gradient Boosting (XGBoost), Kernel Ridge Regression (KRR), Deep Neural Networks (DNN), Support Vector Regression (SVR), and Artificial Neural Networks (ANN) to predict the properties of fluorescent molecules. These models utilized a 128-dimensional *adaptive β-VAE* latent space. All models underwent training, validation, and testing with the same dataset split seed, and they underwent basic hyperparameter optimization. Following this optimization process, Gradient Boosted Regression Trees (GBRT) outperformed the other models, achieving a mean absolute error (MAE) of 0.128 eV and the highest overall performance based on various coefficients. Consequently, GBRT was selected as the final algorithm.

Table S4. Performance of 5-fold cross-validation with standard deviation. To ensure a more robust evaluation, we performed 5-fold cross-validation for 10 iterations using the latent representations learned by the model.

Model	MAE (eV)	RMSE (eV)	R^2	*r*
GBRT	0.134(0.008)	0.217(0.015)	0.719(0.034)	0.850(0.020)
XGBoost	0.151(0.010)	0.230(0.019)	0.681(0.038)	0.828(0.022)
SVR	0.254(0.012)	0.339(0.018)	0.308(0.037)	0.580(0.046)
DNN	0.207(0.028)	0.287(0.017)	0.505(0.078)	0.776(0.008)
ANN(MLP)	0.202(0.028)	0.296(0.028)	0.472(0.113)	0.749(0.022)

Table S5. Performance of various prediction methods for emission energy**.**

Model in this work	MAE (eV)	RMSE (eV)	R^2	*r*
Latent Space from adaptive β-VAE	0.128	0.202	0.74	0.86
Latent Space from optimized AE	0.128	0.203	0.74	0.86
Latent Space from VAE	0.134	0.209	0.72	0.85
SELFIES	0.124	0.195	0.74	0.86
ECFP4 (512)	0.082	0.131	0.88	0.94
**First principal Methods**	**MAE (eV)**			
TD-DFT[9–13]	~0.20			

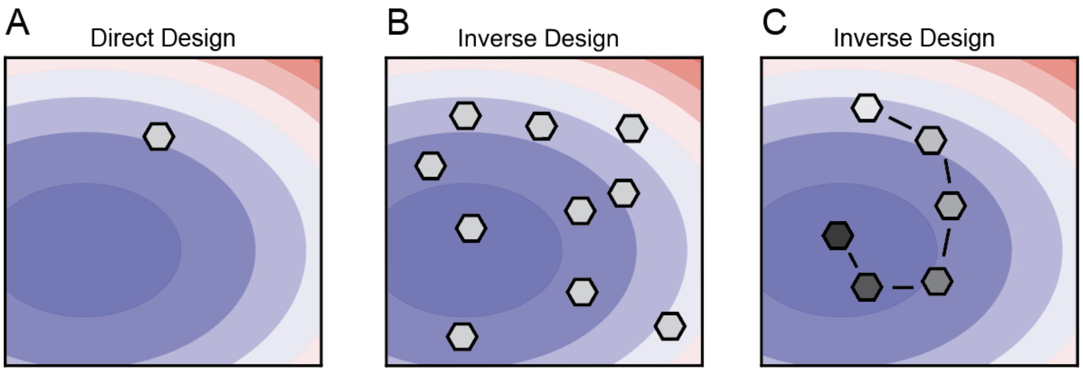

Figure S4. The schematic for various design methods with statistical model. (A) Direct design. (B) Inverse design with arbitrary design. (C) Inverse design with target optimization.

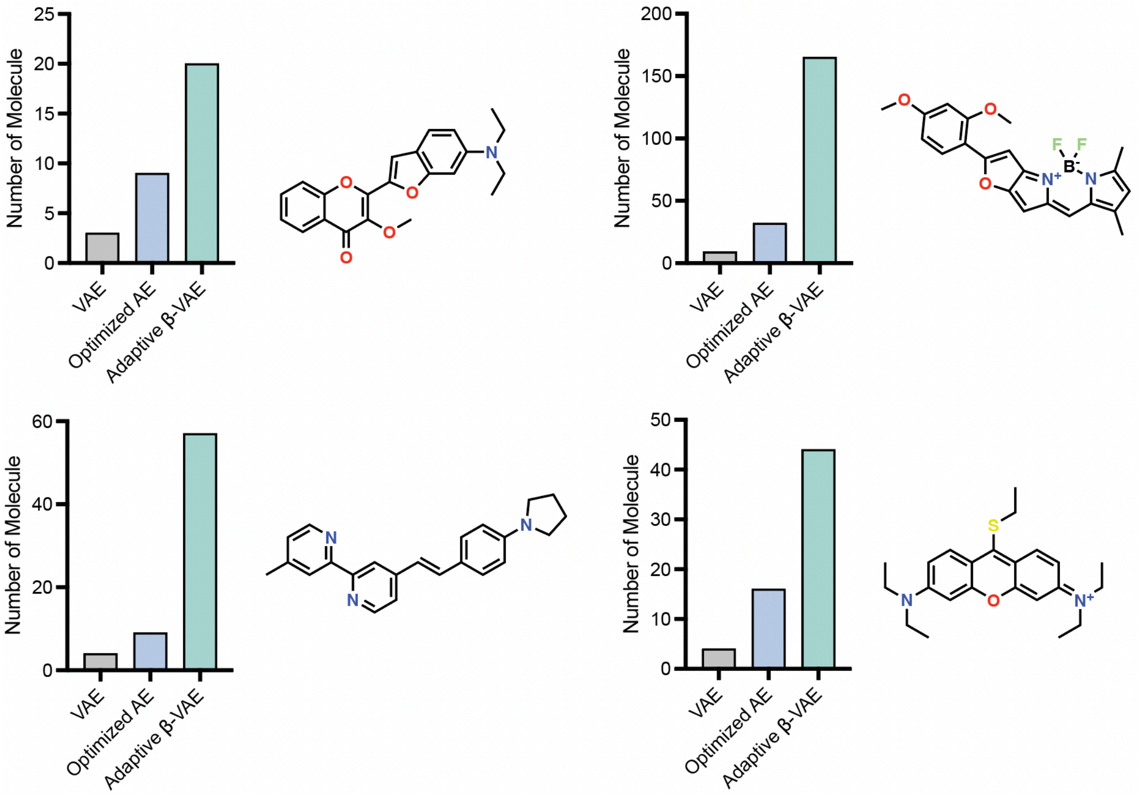

Figure S5. *Adaptive β-VAE* enhance diversity in molecular generation. Numbers of molecules generated from several typical molecules. Structures of the start molecules are shown in figures.

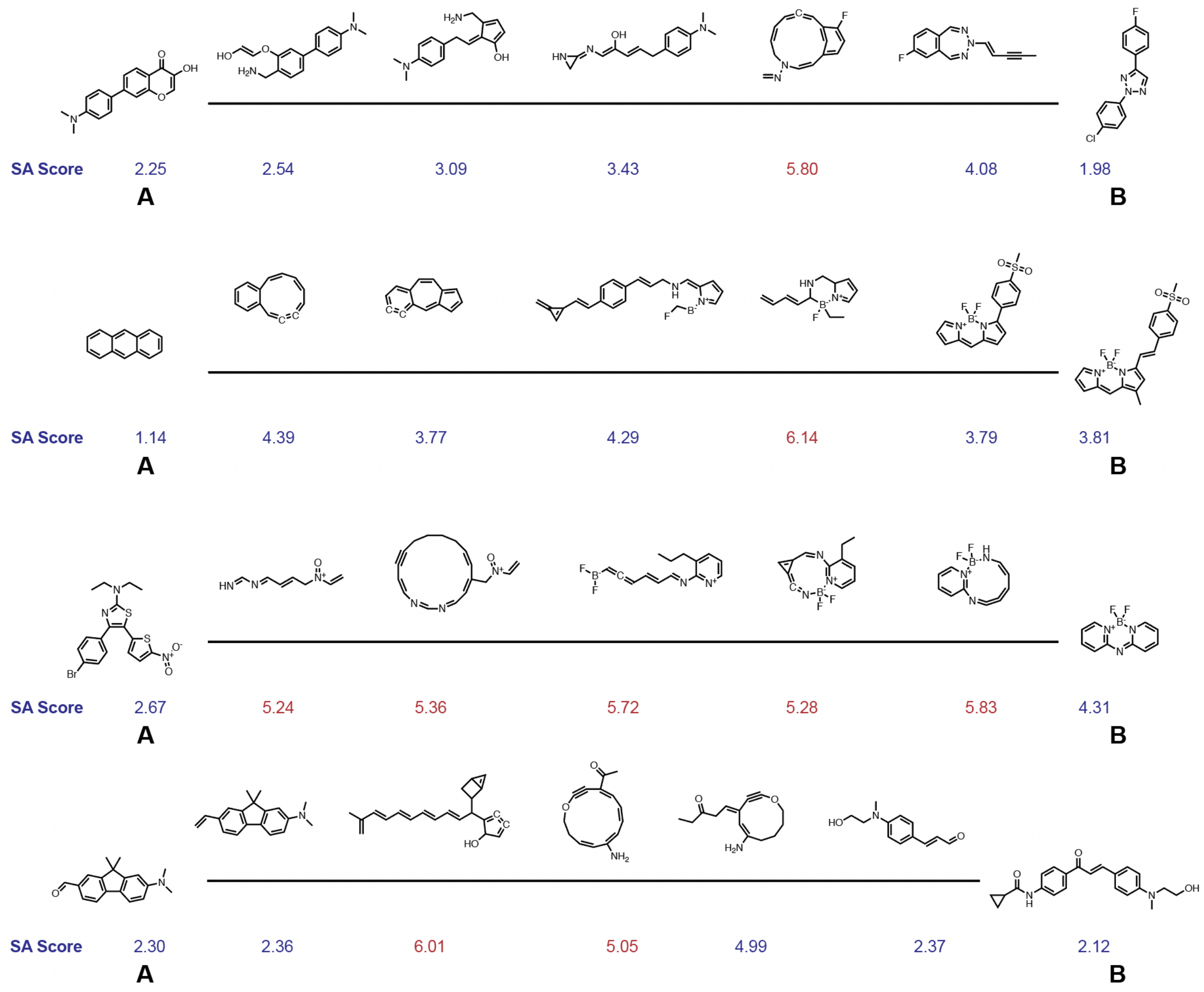

Figure S6. Optimized AE generated viable molecules from latent vector interpolations.

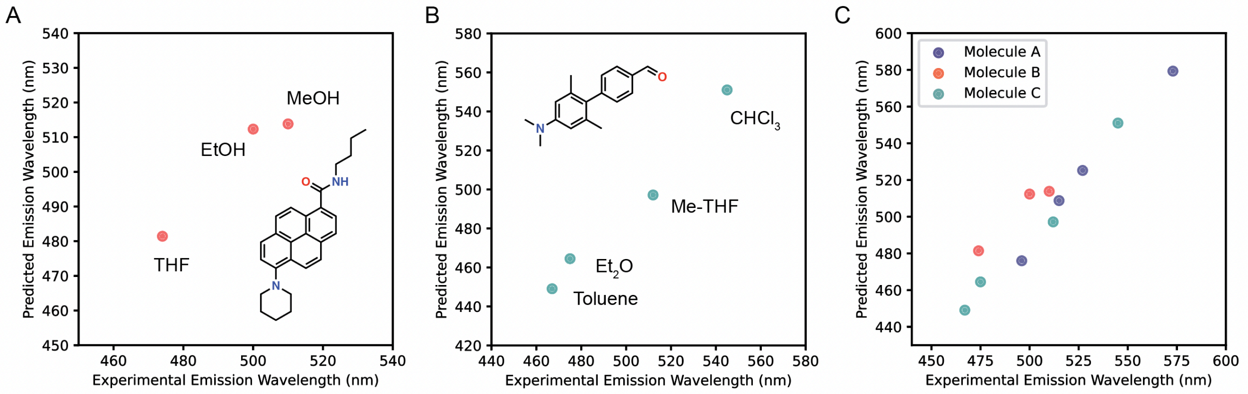

Figure S7. The fluorescence wavelength of typical molecules, (A) molecule B, (B) molecule C in the test set in various solvents. (C) GBRT can reproduce the solvent effects across various molecules.

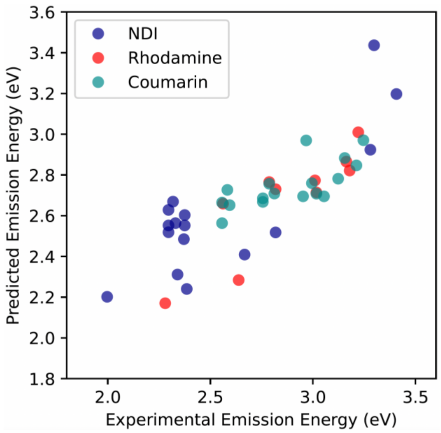

Figure S8. Experimental and predicted emission energy of the selected molecules from the external validation set.

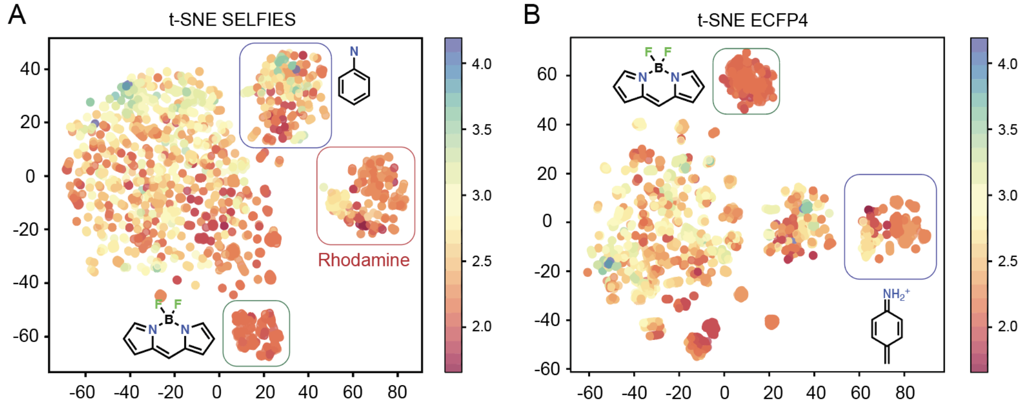

Figure S9. T-distributed stochastic neighbor embedding (t-SNE) of (A) SELFIES and (B) ECFP4. Colors indicate the emission energies.

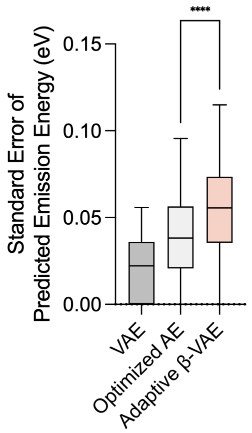

Figure S10. Adaptive β-VAE enhances properties' diversity in molecular generation. The box plot illustrates the ability of adaptive β-VAE to generate viable molecules with varying emission energies from latent vector interpolations.

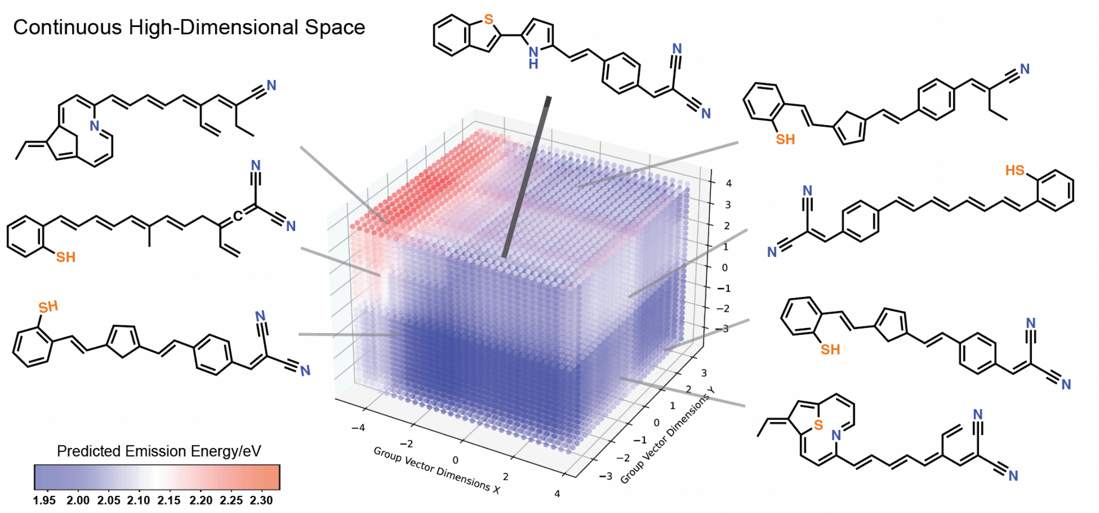

Figure S11. Visualization and analysis of the continuous high-dimensional space obtained by optimized AE, indicating potential for optimization.

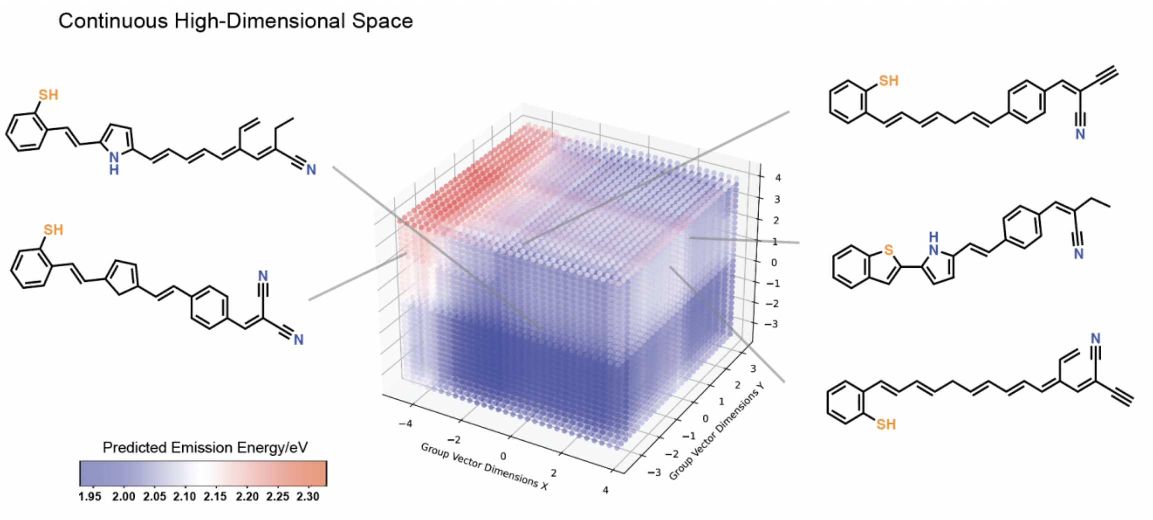

Figure S12. Visualization and analysis of the continuous high-dimensional space from optimized AE for selected molecules with similar backbone, indicating the reliability of predicted value.

3. Protocol for Molecular Synthesis

3.1. General

^{1}H, ^{13}C NMR spectra were acquired on the Bruker 500 MHz NMR spectrometer. ^{1}H NMR and ^{13}C NMR chemical shifts were determined relative to internal standard TMS at δ 0.0 ppm. Chemical shifts (δ) are reported in ppm, and coupling constants (J) are in Hertz (Hz). The following abbreviations were used to explain the multiplicities: s = singlet, d = doublet, t = triplet, q = quartet, m = multiplet.

3.2. General synthetic procedure of 2-arylimidazo[1,2-a]pyridine

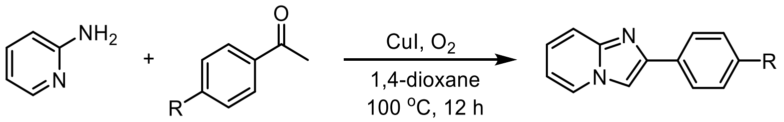

Imidazo[1,2-a]pyridines were prepared according to a procedure reported in the literature.[14] Acetophenone (1.0 mmol), 2-amino pyridine (1.2 mmol), CuI (38 mg, 0.20 mmol) and 1,4-dioxane (15.0 mL) were added to an oven-dried 50 mL round-bottom flask. The resulting solution was stirred at 100 °C for 12 h under ambient air. Upon completion, the solvent was removed under vacuum and the residue was purified by column chromatography to obtain the desired product.

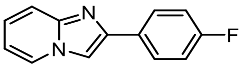

2-(4-fluorophenyl)imidazo[1,2-a]pyridine. Pale-yellow solid; 110 mg, 52% yield. ^{1}H NMR (500 MHz, $CDCl_3$) δ 8.07 (d, J = 6.7 Hz, 1H), 7.96–7.86 (m, 2H), 7.77 (s, 1H), 7.61 (d, J = 9.1 Hz, 1H), 7.20–7.06 (m, 3H), 6.75 (t, J = 6.7 Hz, 1H); ^{13}C NMR (126 MHz, $CDCl_3$) δ 162.7 (d, J = 247.2 Hz), 145.6, 144.9, 130.0 (d, J = 3.5 Hz), 127.7 (d, J = 8.1 Hz), 125.6, 124.8, 117.4, 115.6 (d, J = 21.5 Hz), 112.5, 107.8 ppm.

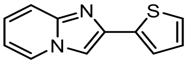

2-(thiophen-2-yl)imidazo[1,2-a]pyridine. Yellow solid; 81 mg, 41% yield. ^{1}H NMR (500 MHz, $CDCl_3$) δ 8.03 (d, J = 6.5 Hz, 1H), 7.73 (s, 1H), 7.58 (d, J = 9.0 Hz, 1H), 7.45 (dd, J = 3.5, 1.0 Hz, 1H), 7.29 (dd, J = 5.0, 0.5 Hz, 1H), 7.13 (m, 1H), 7.07 (dd, J = 5.0, 4.0 Hz, 1H), 6.74 (t, J = 6.5 Hz, 1H); ^{13}C NMR (126 MHz, $CDCl_3$) δ 145.4, 140.8, 137.5, 127.8, 125.5, 125.1, 124.9, 123.7, 117.3, 112.6, 107.5 ppm.

3.3. Synthetic procedure of 3-(methylthio)-5,6-diphenylnaphtho[1',2':4,5]imidazo[1,2-a]pyridine

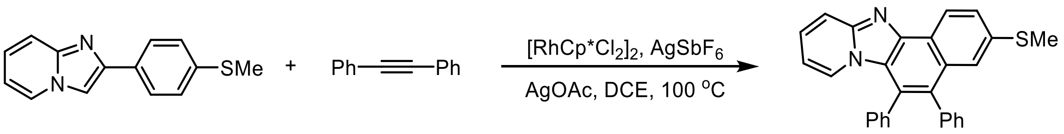

This compound was prepared according to a procedure reported in the literature.[15] 2-(4-(methylthio)phenyl)imidazo[1,2-a]pyridine (72 mg, 0.30 mmol), diphenylethyne (64 mg, 0.36 mmol), $[RhCp^*Cl_2]_2$ (7.4 mg, 4.0 mol %), $AgSbF_6$ (16.5 mg, 16.0 mol%), AgOAc (110 mg, 0.66 mmol), and 1,2-dichloroethane (3.0 mL) were added to a 25 mL Schlenk tube. The reaction mixture was stirred at 100 °C for 12 h. After cooling to room temperature, the solvent was removed

under vacuum and the residue was purified by column chromatography to afford the desired product.

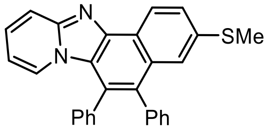

3-(methylthio)-5,6-diphenylnaphtho[1',2':4,5]imidazo[1,2-a]pyridine. Pale-yellow solid; 90 mg, 72% yield. ^{1}H NMR (500 MHz, $CDCl_3$) δ 8.90 (d, J = 8.6 Hz, 1H), 7.84 (d, J = 9.2 Hz, 1H), 7.64 (d, J = 8.5 Hz, 1H), 7.44 (s, 1H), 7.37–7.18 (m, 12H), 6.51 (t, J = 6.9 Hz, 1H), 2.42 (s, 3H). ^{13}C NMR (126 MHz, $CDCl_3$) δ 148.2, 140.9, 138.2, 136.8, 136.4, 132.7, 132.0, 131.6, 130.3, 128.5, 127.8, 127.6, 127.4, 127.3, 126.7, 126.4, 125.5, 124.1, 123.7, 123.45, 122.44, 117.71, 110.68, 15.88 ppm.

3.4. Synthetic procedure of 4-(benzo[d]oxazol-2-yl)benzonitrile

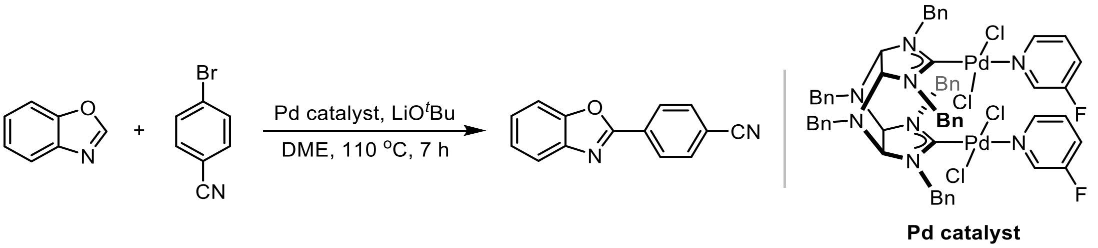

This compound was prepared according to a procedure reported in the literature.[16] Under an argon atmosphere, benzoxazole (143 mg, 1.20 mmol), ^{t}BuOLi (120 mg, 1.50 mmol), 4-bromobenzonitrile (182 mg, 1.00 mmol), Pd catalyst (6.5 mg, 0.50 mol%), and DME (4.0 mL) were added to a 25 mL Schlenk tube. The mixture was stirred at 110 °C for 5 h. Upon completion, the mixture was transferred to a separation funnel, ethyl acetate (10 mL) was added. The pH of the mixture was adjusted to neutral by addition of dilute aqueous HCl solution. The mixture was then washed with water (10 mL × 3) and brine (10 mL). The organic layer was dried over anhydrous Na_2SO_4, filtered and the solvent was removed under vacuum. The residue was purified by column chromatography to afford the desired product.

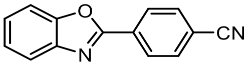

4-(benzo[d]oxazol-2-yl)benzonitrile. Yellow solid, 205 mg, 88% yield. ^{1}H NMR (500 MHz, CD_2Cl_2) δ 8.28 (d, J = 8.2, 2H), 7.75 (d, J = 8.2 Hz, 2H), 7.71 (m, 1H), 7.56 (d, J = 8.5 Hz, 1H), 7.38–7.30 (m, 2H); ^{13}C NMR (126 MHz, CD_2Cl_2) δ 161.5, 151.4, 142.4, 133.2, 131.5, 128.3, 126.5, 125.4, 120.8, 118.6, 115.1, 111.2 ppm.

3.5. NMR spectrum for synthesized molecules

^{1}H NMR spectrum of 4-(benzo[d]oxazol-2-yl)benzonitrile

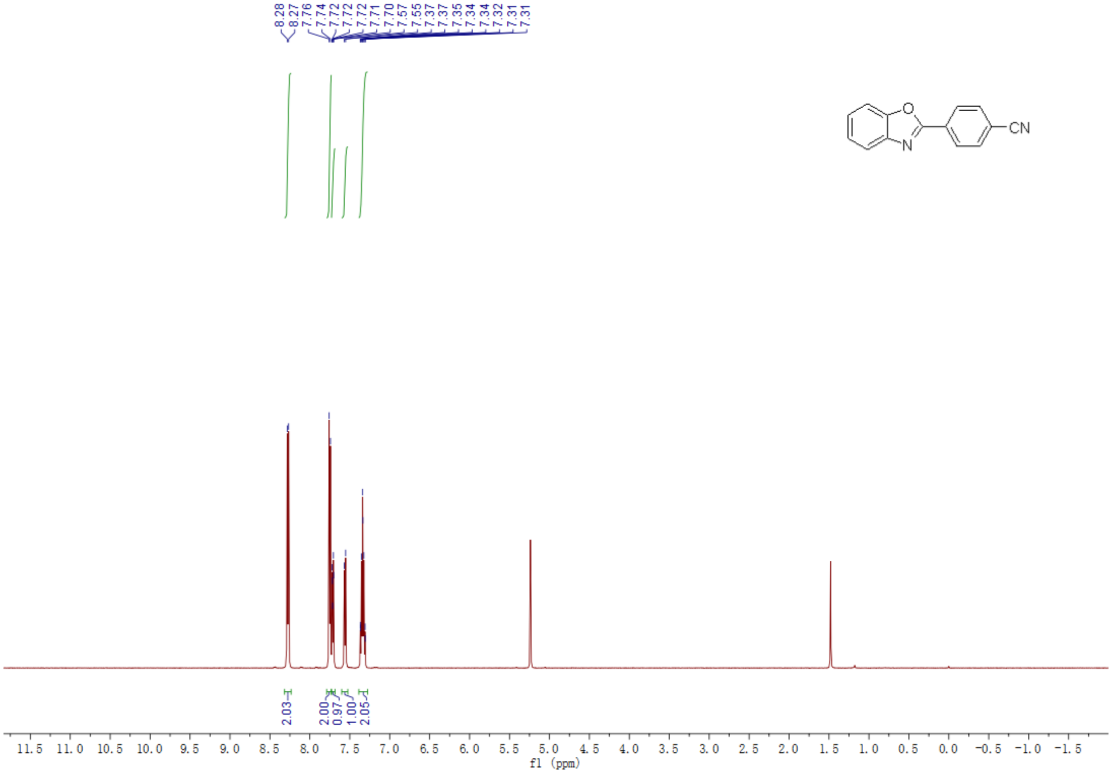

^{13}C NMR spectrum of 4-(benzo[d]oxazol-2-yl)benzonitrile

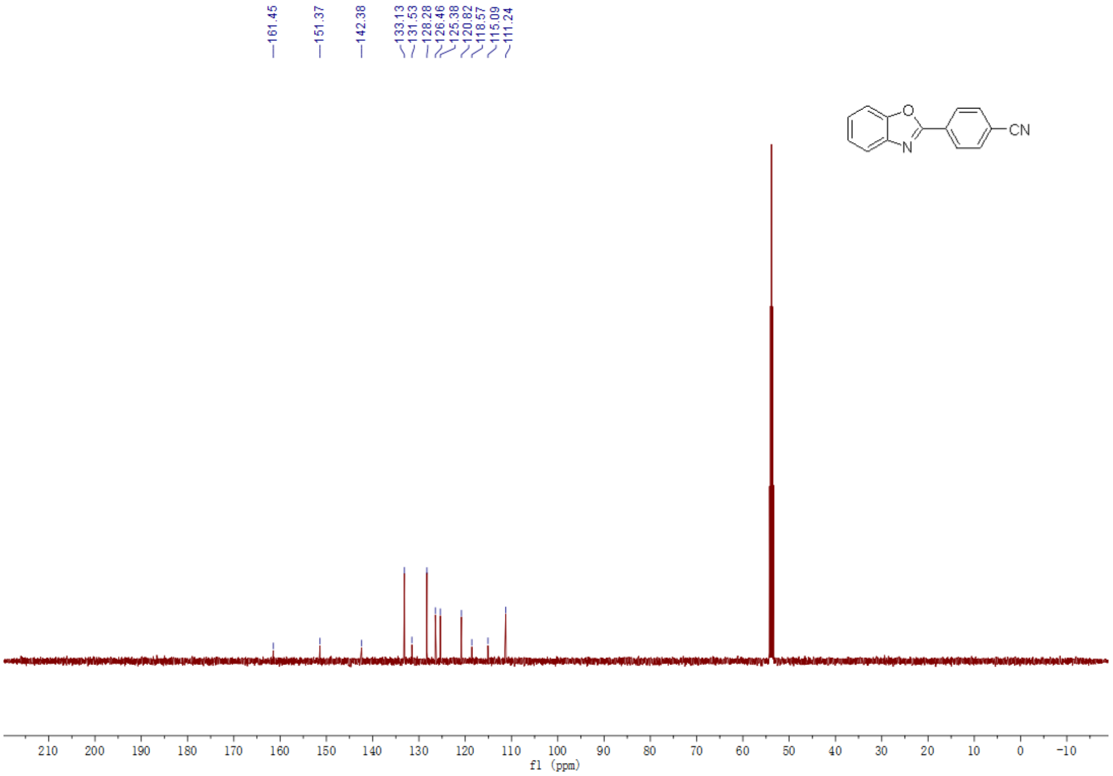

^{1}H NMR spectrum of 2-(thiophen-2-yl)imidazo[1,2-a]pyridine

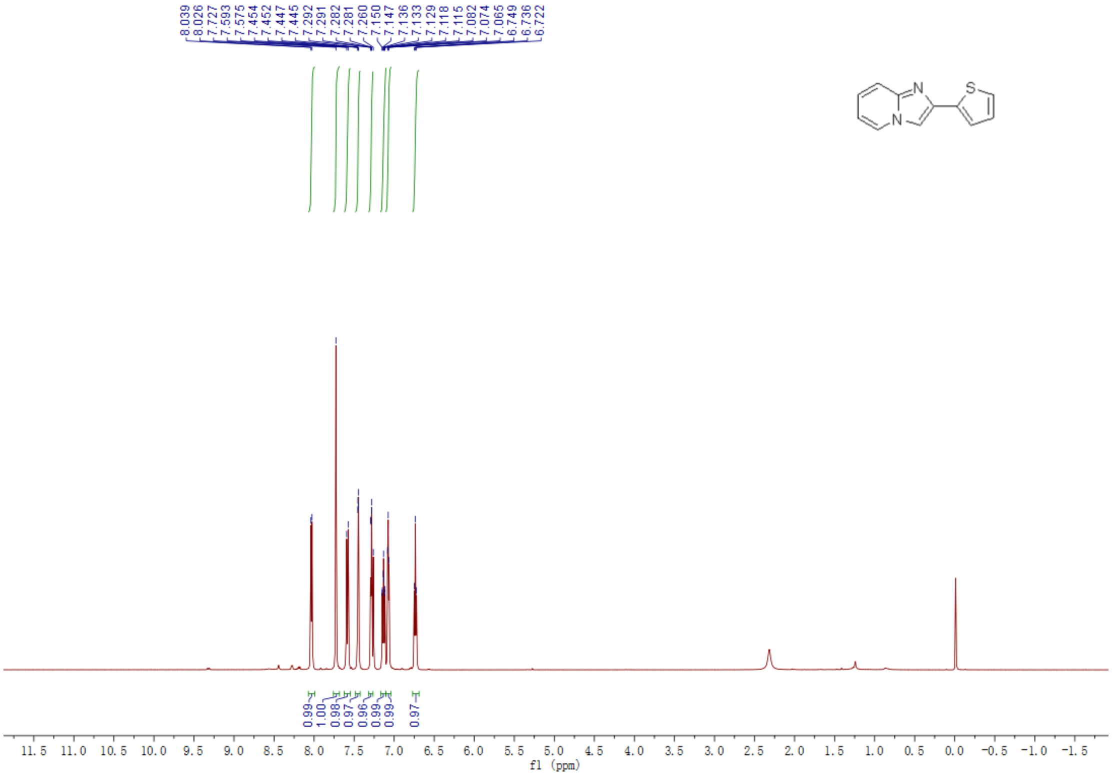

^{13}C NMR spectrum of 2-(thiophen-2-yl)imidazo[1,2-a]pyridine

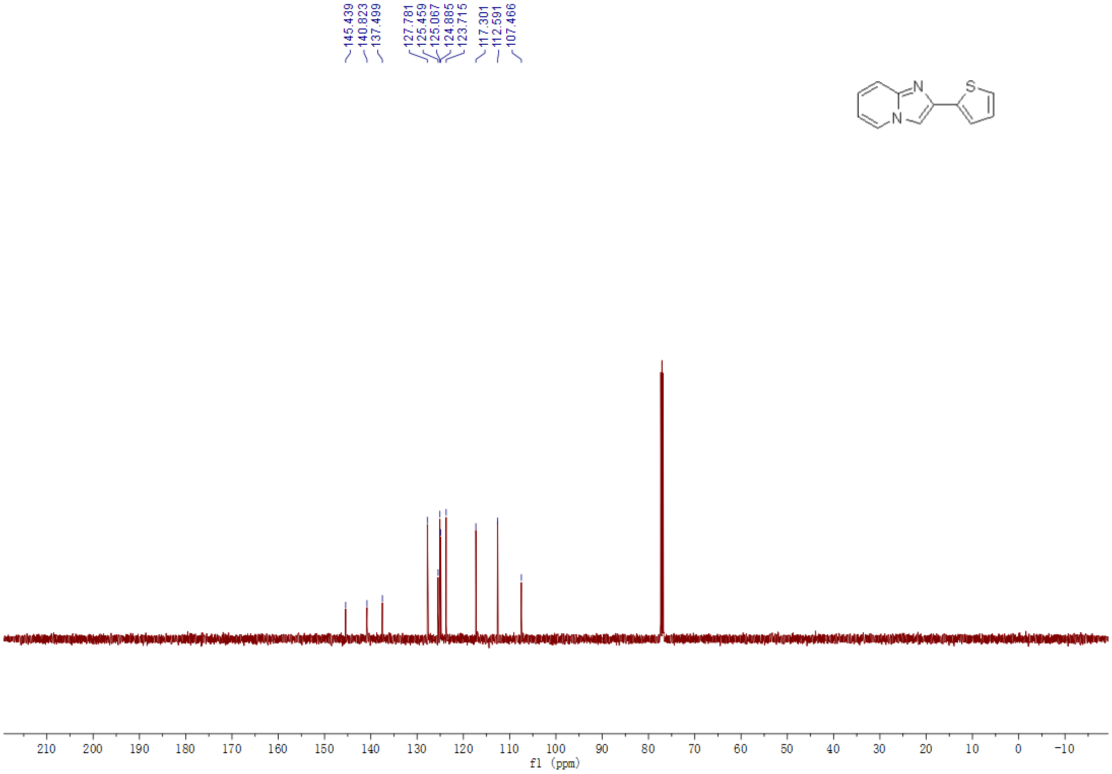

^{1}H NMR spectrum of 2-(4-fluorophenyl)imidazo[1,2-a]pyridine

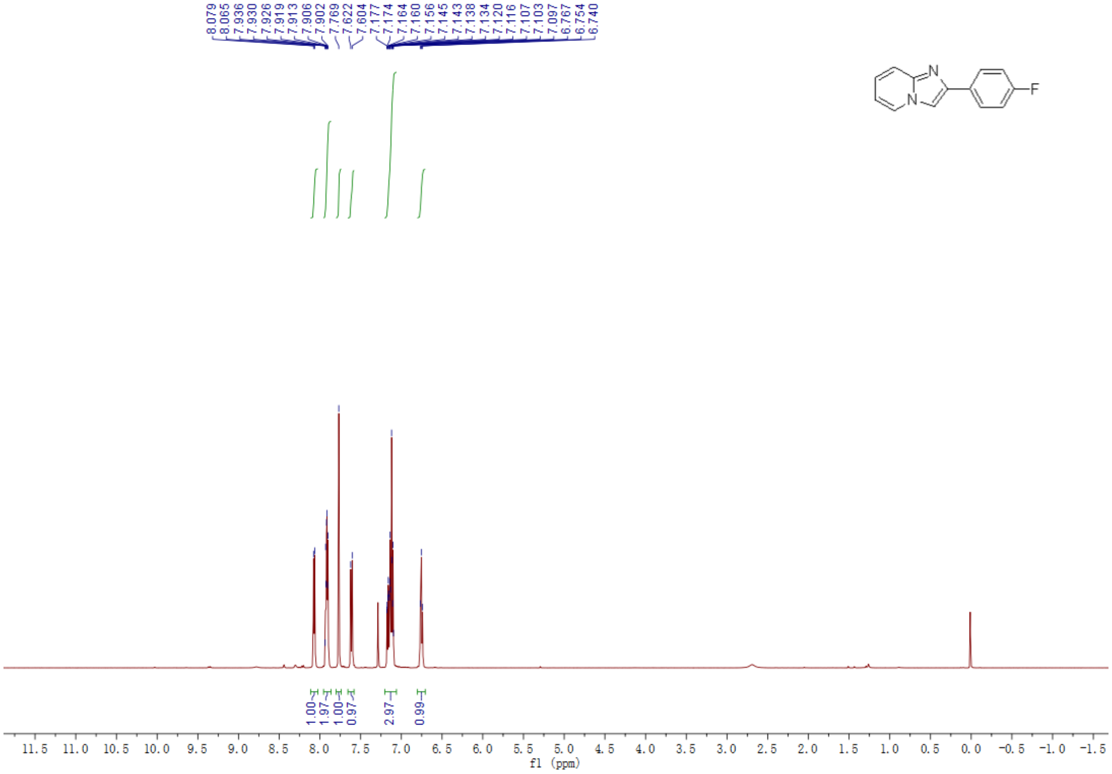

^{13}C NMR spectrum of 2-(4-fluorophenyl)imidazo[1,2-a]pyridine

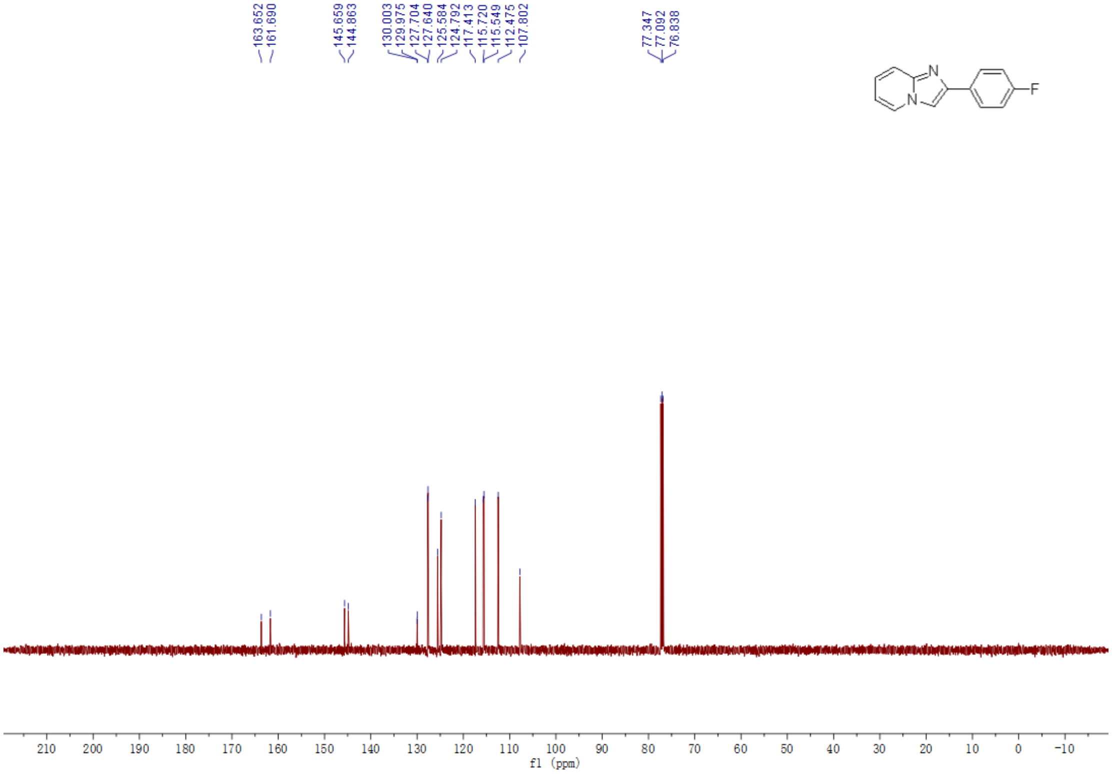

^{1}H NMR spectrum of 3-(methylthio)-5,6-diphenylnaphtho[1',2':4,5]imidazo[1,2-a]pyridine

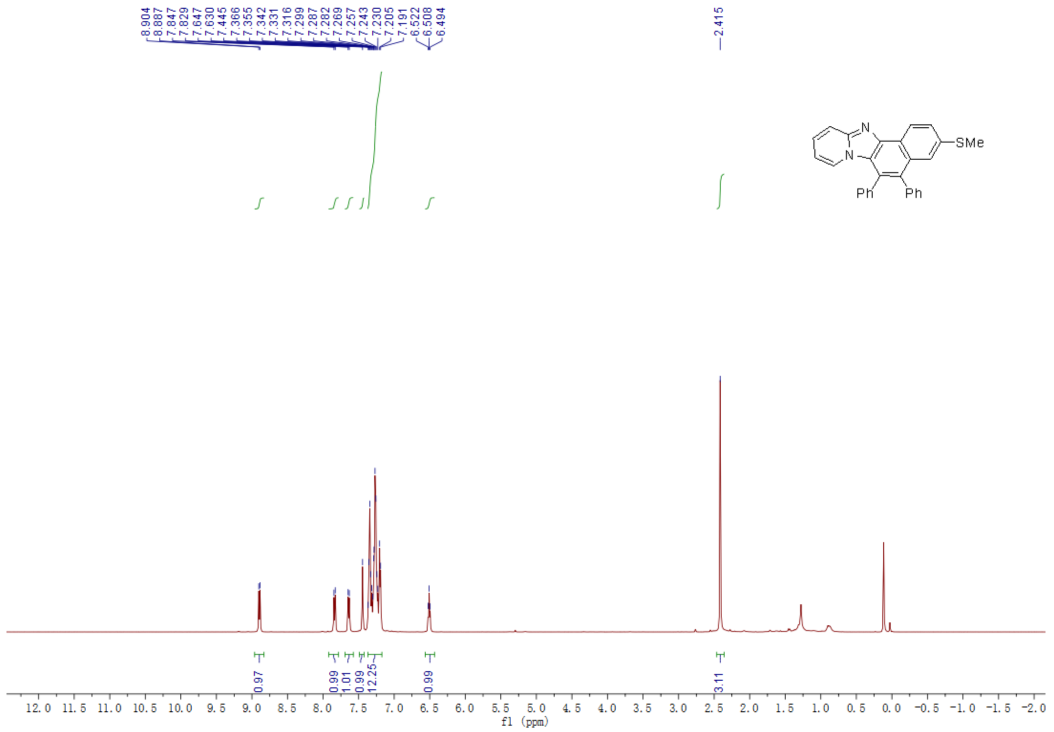

^{13}C NMR spectrum of 3-(methylthio)-5,6-diphenylnaphtho[1',2':4,5]imidazo[1,2-a]pyridine

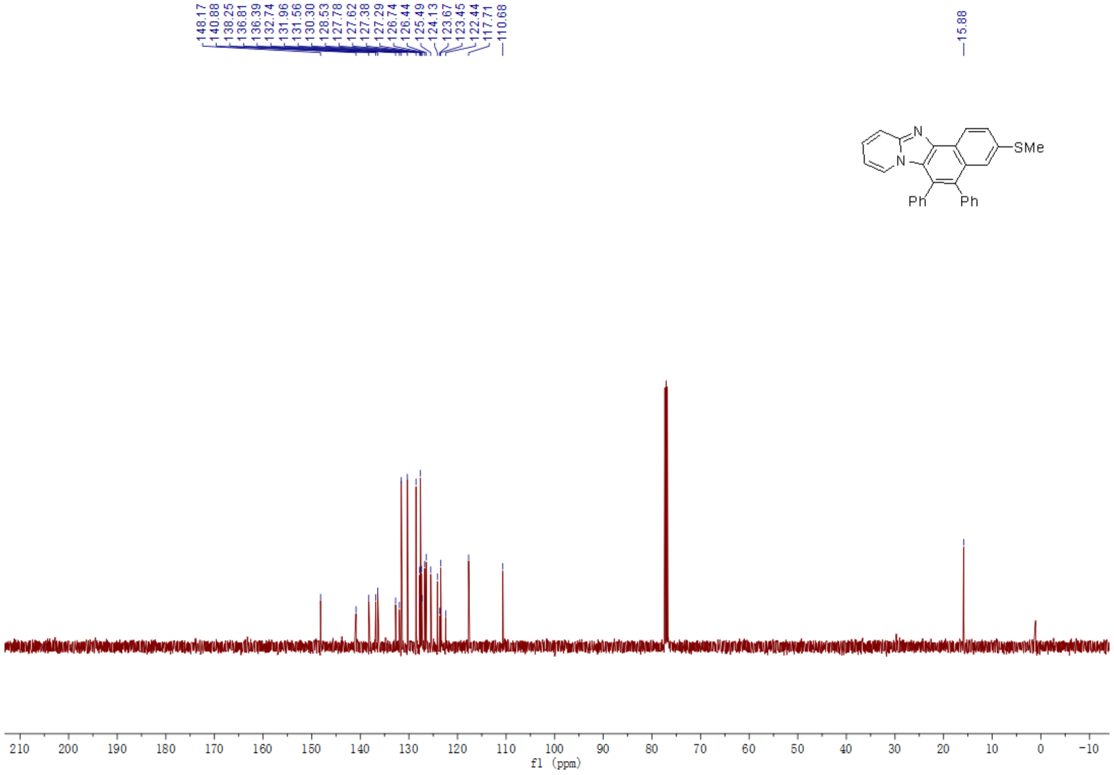

4. Reference

(1) Kingma, D. P.; Welling, M. Auto-Encoding Variational Bayes. arXiv December 10, 2022. https://doi.org/10.48550/arXiv.1312.6114.

(2) Ramakrishnan, R.; Dral, P. O.; Rupp, M.; von Lilienfeld, O. A. Quantum Chemistry Structures and Properties of 134 Kilo Molecules. *Sci. Data* **2014**, *1* (1), 140022. https://doi.org/10.1038/sdata.2014.22.

(3) Ju, C.-W.; Bai, H.; Li, B.; Liu, R. Machine Learning Enables Highly Accurate Predictions of Photophysical Properties of Organic Fluorescent Materials: Emission Wavelengths and Quantum Yields. *J. Chem. Inf. Model.* **2021**, *61* (3), 1053–1065. https://doi.org/10.1021/acs.jcim.0c01203.

(4) Gómez-Bombarelli, R.; Wei, J. N.; Duvenaud, D.; Hernández-Lobato, J. M.; Sánchez-Lengeling, B.Sheberla, D.; Aguilera-Iparraguirre, J.; Hirzel, T. D.; Adams, R. P.; Aspuru-Guzik, A. Automatic Chemical Design Using a Data-Driven Continuous Representation of Molecules. ACS Cent. Sci. 2018, 4 (2), 268–276. https://doi.org/10.1021/acscentsci.7b00572.

(5) Prettenhofer, P.; Louppe, G. Gradient Boosted Regression Trees in Scikit-Learn; 2014.

(6) Natekin, A.; Knoll, A. Gradient Boosting Machines, a Tutorial. *Front. Neurorobotics* **2013**, *7*.

(7) Krenn, M.; Häse, F.; Nigam, A.; Friederich, P.; Aspuru-Guzik, A. Self-Referencing Embedded Strings (SELFIES): A 100% Robust Molecular String Representation. *Mach. Learn. Sci. Technol.* **2020**, *1* (4), 045024. https://doi.org/10.1088/2632-2153/aba947.

(8) Bannwarth, C.; Ehlert, S.; Grimme, S. GFN2-xTB—An Accurate and Broadly Parametrized Self-Consistent Tight-Binding Quantum Chemical Method with Multipole Electrostatics and Density-Dependent Dispersion Contributions. *J. Chem. Theory Comput.* **2019**, *15* (3), 1652–1671. https://doi.org/10.1021/acs.jctc.8b01176.

(9) Laurent, A. D.; Jacquemin, D. TD-DFT Benchmarks: A Review. *Int. J. Quantum Chem.* **2013**, *113* (17), 2019–2039. https://doi.org/10.1002/qua.24438.

(10)Ju, C.-W.; French, E. J.; Geva, N.; Kohn, A. W.; Lin, Z. Stacked Ensemble Machine Learning for Range-Separation Parameters. *J. Phys. Chem. Lett.* **2021**, *12* (39), 9516–9524. https://doi.org/10.1021/acs.jpclett.1c02506.

(11)Chantzis, A.; Cerezo, J.; Perrier, A.; Santoro, F.; Jacquemin, D. Optical Properties of Diarylethenes with TD-DFT: 0–0 Energies, Fluorescence, Stokes Shifts, and Vibronic Shapes. *J. Chem. Theory Comput.* **2014**, *10* (9), 3944–3957. https://doi.org/10.1021/ct500371u.

(12)Charaf-Eddin, A.; Planchat, A.; Mennucci, B.; Adamo, C.; Jacquemin, D. Choosing a Functional for Computing Absorption and Fluorescence Band Shapes with TD-DFT. *J. Chem. Theory Comput.* **2013**, *9* (6), 2749–2760. https://doi.org/10.1021/ct4000795.

(13)Hall, D.; Sancho-García, J. C.; Pershin, A.; Beljonne, D.; Zysman-Colman, E.; Olivier, Y. Benchmarking DFT Functionals for Excited-State Calculations of Donor–Acceptor TADF Emitters: Insights on the Key Parameters Determining Reverse Inter-System Crossing. *J. Phys. Chem. A* **2023**, *127* (21), 4743–4757. https://doi.org/10.1021/acs.jpca.2c08201.

(14)Kaswan, P.; Pericherla, K.; Saini, H. K.; Kumar, A. One-Pot, Three Component Tandem Reaction of 2-Aminopyridines, Acetophenones and Aldehydes: Synthesis of 3-Aroylimidazo[1,2-a]Pyridines. *RSC Adv.* **2014**, *5* (5), 3670–3677. https://doi.org/10.1039/C4RA13056A.

(15)Qi, Z.; Yu, S.; Li, X. Rh(III)-Catalyzed Oxidative Annulation of 2-Phenylimidazo[1,2-a]Pyridines with Alkynes: Mono versus Double C-H Activation. *J. Org. Chem.* **2015**, *80* (7), 3471–3479. https://doi.org/10.1021/acs.joc.5b00059.

(16) Jiang, W.; Huang, W.; Xu, M.; Leng, X.; Lu, L.; Shen, Q. Diimidazolium Salt HBDIM: An Easily Available, Low-Cost, CageCarbene Precursor with Broad Applications in Transition Metal-Catalyzed Reactions. *Chem. – Eur. J.* **2023**, *29* (40), e202300991. https://doi.org/10.1002/chem.202300991.
